# Dietary *S. maltophilia* induces supersized lipid droplets by enhancing lipogenesis and ER-LD contacts in *C. elegans*

**DOI:** 10.1080/19490976.2021.2013762

**Published:** 2022-02-03

**Authors:** Kang Xie, Yangli Liu, Xixia Li, Hong Zhang, Shuyan Zhang, Ho Yi Mak, Pingsheng Liu

**Affiliations:** a National Laboratory of Biomacromolecules, Cas Center for Excellence in Biomacromolecules, Institute of Biophysics, Chinese Academy of Sciences, Beijing, China; b University of Chinese Academy of Sciences, Beijing, China; c Division of Life Science, The Hong Kong University of Science and Technology, Hong Kong, China

**Keywords:** *Stenotrophomonas maltophilia*/obesity/dhs-3/mdt-28/lipid droplet

## Abstract

Dietary and symbiotic bacteria can exert powerful influence on metazoan lipid metabolism. Recent studies have emerged that microbiota have a role in animal obesity and related health disorders, but the mechanisms by which bacteria influence lipid storage in their host are unknown. To reduce the complexity of the relationship between gut microbiota and the host, *Caenorhabditis elegans* (*C. elegans*) has been chosen as a model organism to study interspecies interaction. Here, we demonstrate that feeding *C. elegans* with an opportunistic pathogenic bacterium *Stenotrophomonas maltophilia* (*S. maltophilia*) retards growth and promotes excessive neutral lipid storage. Gene expression analysis reveals that dietary *S. maltophilia* induces a lipogenic transcriptional response that includes the SREBP ortholog SBP-1, and fatty acid desaturases FAT-6 and FAT-7. Live imaging and ultrastructural analysis suggest that excess neutral lipid is stored in greatly expanded lipid droplets (LDs), as a result of enhanced endoplasmic reticulum (ER)-LD interaction. We also report that loss of function mutations in *dpy-9* in *C. elegans* confers resistance to *S. maltophilia*. Dietary *S. maltophilia* induces supersized LDs by enhancing lipogenesis and ER-LD contacts in *C. elegans*. This work delineates a new model for understanding microbial regulation of metazoan physiology.

## Introduction

In the wild, the nematode *C. elegans* feeds on a variety of soil bacteria including *Pseudomonas mendocina, Bacillus megaterium, Comamonas sp*.^1–6^ In contrast, the *E. coli* OP50 is commonly used as the standard laboratory diet.^7,8^ Therefore, the full range of physiological response in *C. elegans* toward dietary bacteria remains obscure. Nevertheless, increasing evidence suggests that *C. elegans* is capable of integrating olfactory, mechanical and nutritional cues to identify its preferred bacterial diet. Such dietary choices can subsequently exert significant impact on *C. elegans* lifespan and metabolism.^9–18^

Lipid droplets (LDs) are conserved membrane-bound organelles for cellular neutral lipid storage. Over the last decade, to reveal LD conservation from bacteria to humans, our laboratory has focused on using mass spectrometry to determine the proteome of isolated LDs from a wide range of uni- and multi-cellular organisms, including *C. elegans*. Accordingly, we identified three major LD resident proteins in *C. elegans*, DHS-3, MDT-28, and F22F7.1.^19–21^ When expressed as fluorescent fusion proteins in transgenic worms, DHS-3 and MDT-28 serve as faithful markers for monitoring LD morphology in live animals. Importantly, we found a strong correlation between LD size and number with organismal neutral lipid storage.

Metabolic syndromes are in fact a lipid storage disorder, ectopic lipid storage that originates from obesity. Obesity is a symptom of excessive accumulation of neutral lipids such as triacylglycerol (TAG) in white adipocytes. Adipose tissue governs the individual’s lipid homeostasis at the tissue level. However, at the cellular level, TAG is stored in LDs. The distribution of LDs in non-adipose tissues, the size and number of LDs in these cells, as well as the type and amount of fatty acids in TAG, are the key factors of metabolic disorders. In white adipose tissue, adipocytes contain unilocular lipid droplets with diameter between 50 μm and 150 μm and their cytoplasm only takes less than 15% of cell volume. Thereby, the LD size is typically considered synonymous for the adipocyte size.^22,23^ In other cells, such as liver cells, the number and size of LDs vary with the state of the cell.^24–26^ Therefore, morphological study of lipid droplets is of great significance for understanding obesity and ectopic lipid storage.

Here we apply our suite of fluorescence markers for the ER and LDs, to study the effect of an environmental bacterium *S. maltophilia* on neutral lipid storage in *C. elegans*. Interestingly, clinical isolates of *S. maltophilia* have been regarded as pathogenic in immunocompromised humans, but not necessarily to nematodes.^27–29^ We found that *C. elegans* grew slower and accumulated significantly more triacylglycerol (TAG) when fed *S. maltophilia*, instead of the standard laboratory diet, *E. coli* OP50. By genetic analysis, we uncovered a transcriptional regulatory network that promoted lipogenesis in response to *S. maltophilia*. Coupled with the remodeling of ER-LD interaction, massive LD expansion ensued. Accordingly, attenuation of ER-LD interaction partially suppressed the effect of dietary *S. maltophilia* on neutral lipid storage. Our results help establishing a new *C. elegans*-microbe experimental paradigm for the study of dietary factors that modulate lipid metabolism.

## Results

### Size of lipid droplet in C. elegans is regulated by dietary microbe

**Figure 1.**
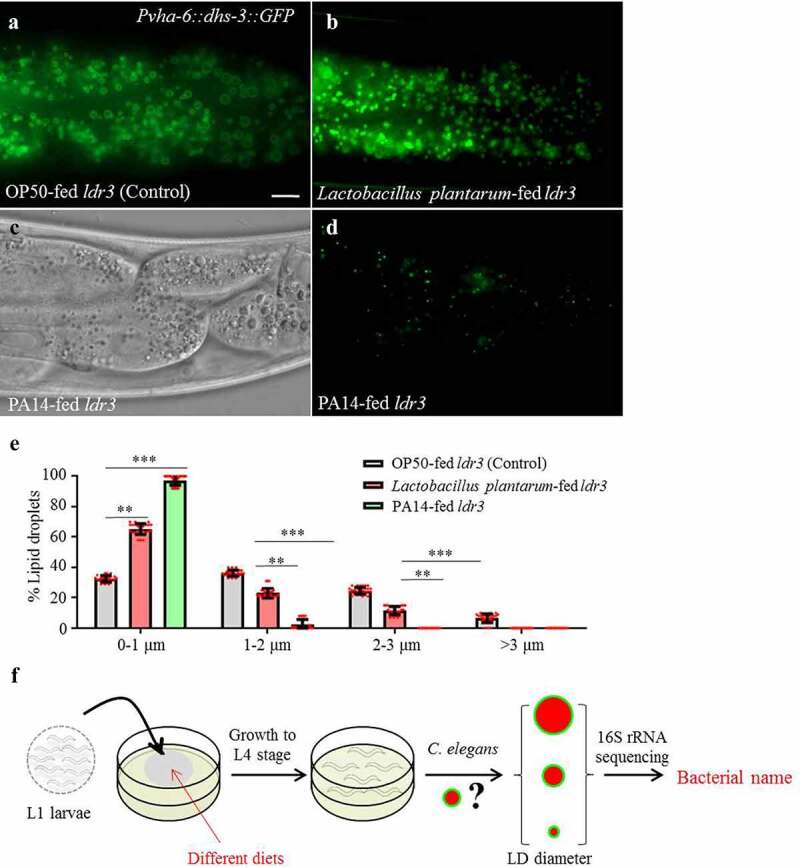
***Lactobacillus plantarum* and PA14 reduce the size of LDs in *C. elegans***. (a) Fluorescence micrographs of LDs in the intestine of OP50-fed worms. Scale bar = 5 μm. (b) Fluorescence micrographs of *Pvha-6::dhs-3::GFP* in *Lactobacillus plantarum-*fed worms. (c) Differential interference contrast (DIC) images of LDs in PA14-fed worms. (d) Fluorescence micrographs of *Pvha-6::dhs-3::GFP* in PA14-fed worms. (e) Distribution of the LD size (% Lipid droplets), as measured from DHS-3::GFP labeled worms from (a, b, d). Distribution of the LD size was measured for least 10 animals from at least three biological replicates. Data are shown as mean ± SEM, student’s *t*-test, **P < .01, ***P < .001, n = 30. (f) Schematic representation of the screening method to identify bacteria that affect host LDs.

Two bacterial species were previously shown to reduce *C. elegans* lipid accumulation.^30,31^ To recapitulate these results, we fed *Lactobacillus plantarum* and *Pseudomonas aeruginosa* (strain UCBPP-PA14, PA14) to transgenic worms that express the intestinal LD marker, DHS-3::GFP (Figure 1a-1d). We found that *Lactobacillus plantarum* and *Pseudomonas aeruginosa* reduced the average LD diameter by 26.7% and 83.3%, respectively (Figure 1a-e). We were therefore encouraged to use our DHS-3::GFP and MDT-28::mCherry reporter strains for further exploration of the effect of bacterial diets on *C. elegans* neutral lipid storage.^9^

We performed a small-scale screen to identify bacteria that modulate *C. elegans* lipid metabolism. To capture environmental bacteria, Nematode Growth Medium (NGM) plates were left open in the laboratory. Bacteria that yielded individual colonies were isolated, cultured in liquid medium, and subsequently re-introduced to NGM plates to form single-species bacterial lawns. Synchronized L1 stage transgenic reporter worms that express DHS-3::GFP were allowed to develop with the environmental bacteria as food. We subsequently quantified the LD size in larval L4 stage worms. Bacteria that significantly altered LD size of worms were taken through multiple cloning steps to obtain a pure clone. The cultures were then fed again to worms to verify the effect and the bacteria were identified by 16S rRNA sequencing (Figure 1f). Worms fed these positive clones invariably showed growth retardation or arrest (Table 1). We then focused on *S. maltophilia* because it was the only bacterial species so far tested that significantly increased the LD size of worms.

**Table 1.** The effects of different bacterial diets on *C. elegans* growth and LD size

Bacterial strain	Negative or postive	Nematode growth status	The diamater of top 10 LDs (μm)
*Escherichia coli*(OP50)	gram-negative	control	1.97
*Escherichia coli* (HT115)	gram-negative	slightly slow	1.81
*Ochrobactrum thiophenivorans*	gram-negative	slow	1.87
*Pseudomonas aeruginosa* (PA14)	gram-negative	arrest	0.35
*Stenotrophomonas maltophilia*	gram-negative	slow	5.85
*Carboxylicivirga mesophila*	– – – –	arrest	0.57
*Xanthomonas campestris pv. campestris*	gram-negative	slow	1.82
*X. oryzae pv. oryzae*	gram-negative	slow	1.75
*Listeria monocytogenes*	gram-negative	arrest	1.42
*Staphylococcus aureus*	gram-negative	slow	1.36
*Bacillus subtilis*	gram-negative	slow	1.79

### S. maltophilia promotes lipid droplet expansion in C. elegans

**Figure 2.**
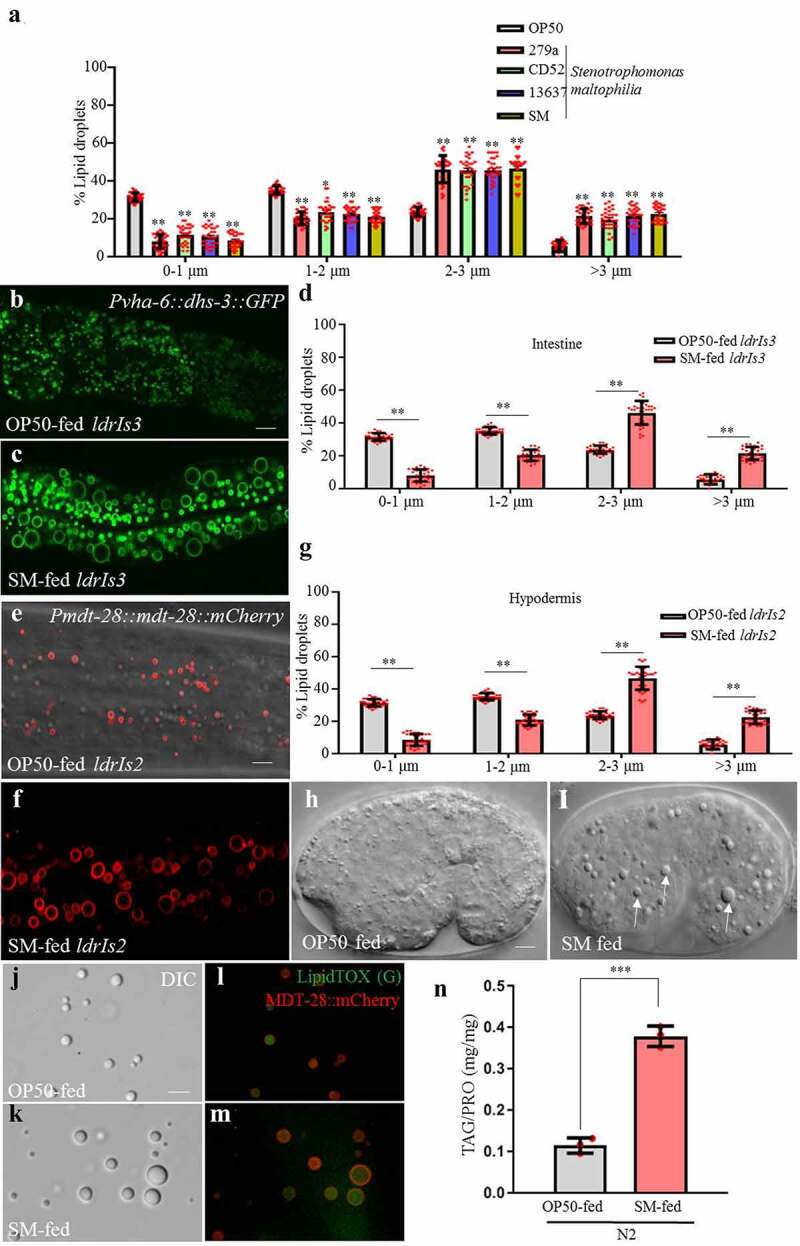
***S. maltophilia* induces supersized LDs in *C. elegans.*** (a) Distribution of the LD size (% LDs), as measured from *ldrIs3* fed different *S. maltophilia* strains. Distribution of the LD size was measured for least 10 animals from at least three biological replicates. Data are shown as mean ± SEM, student’s *t*-test, *P < .05, **P < .01, n = 30. (b) Fluorescence micrographs of LDs in the intestine of OP50-fed worms. Scale bar = 5 μm. (c) Fluorescence micrographs of *Pvha-6::dhs-3::GFP* in *S. maltophilia-*fed worms. (d) Distribution of the LD size (% lipid droplets), as measured from *ldrIs3* fed by OP50 and by *S. maltophilia* strains from (b and c). Distribution of the LD size was measured for least 10 animals from at least three biological replicates. Data are shown as mean ± SEM, student’s *t*-test, **P < .01, n = 30. (e) Fluorescence micrographs of LDs in the hypodermis of OP50-fed worms. Scale bar = 5 μm. (f) Fluorescence micrographs of *Pmdt-28::mdt-28::mCherry* labeled LDs in *S. maltophilia-*fed worms. (g) Distribution of the LD size (% lipid droplets), as measured from *ldrIs2* fed OP50 and *S. maltophilia* strains from (e and f). Data are presented as mean ± SD of 10 animals for each worm strain, the assay was repeated three times, student’s *t*-test, **P < .01, n = 30. (h) DIC images of LDs in the embryonic stage of OP50-fed worms. Scale bar = 5 μm. (i) DIC images of LDs in the embryonic stage of *S. maltophilia*-fed worms. The white arrows point to the *S. maltophilia*-enlarged LDs. (j) DIC images of LDs isolated from transgenic animals expressing MDT-28::mCherry and fed by OP50, the worms were cultured from L1 to L4 stage. Scale bar = 5 μm. (k) DIC images of LDs isolated from transgenic animals expressing MDT-28::mCherry and fed by *S. maltophilia*. (l) Fluorescence micrographs of LDs isolated from transgenic animals expressing MDT-28::mCherry and fed by OP50, with LipidTOX Green stained-LDs and MDT-28::mCherry labeled LDs signals merged. (m) Fluorescence micrographs of LDs isolated from transgenic animals expressing MDT-28::mCherry and fed by *S. maltophilia*, with LipidTOX Green stained-LDs and MDT-28::mCherry labeled LDs signals merged. (n) Comparison of TAG levels between OP50 and *S. maltophilia*-fed N2 worms, the assay was repeated three times, student *t*-test, *** *P* < .001, n = 3.

The bacterium *S. maltophilia* induced a striking increase in LD diameter after 2 days of feeding (Table 1). To confirm the effect, we repeated the experiment using *S. maltophilia* strains isolated in other laboratories^32,33^ (Table 2). Similar to our original isolate, all strains tested reproducibly promoted LD expansion in worms (Figure 2a). Our results suggest that *S. maltophilia* harbors a species-specific factor that potently modulates neutral lipid storage in *C. elegans*.

**Table 2.** *S. maltophilia* strains

Strain name	Bacterial source
*Stenotrophomonas maltophilia 279a*	ATCC
*Stenotrophomonas maltophilia CD52*	Keqin Zhang’s lab
*Stenotrophomonas maltophilia 13637*	Wei Qian’s lab
*Stenotrophomonas maltophilia* (s)	Pingsheng Liu’s lab

Next, we asked if the effect of *S. maltophilia* feeding on LD size was restricted to *C. elegans* intestine. As indicated by the *vha-6* promoter driven, intestine-specific DHS-3::GFP marker, the LD diameter distribution of worms fed *S. maltophilia* had an increased percentage of larger LDs (>2 μm), but a decreased percentage of smaller LDs (<2 μm) compared to those of WT worms (Figure 2b-d). To ensure the reliability of the results of DHS-3::GFP, DGAT-2::GFP was used for further verification. The results showed that *S. maltophilia* feeding also induced DGAT-2::GFP-labeled LD expansion (Fig. S9). To determine the change of LD size in the hypodermis, we used MDT-28::mCherry that was expressed under the control of the *mdt-28* promoter (Figure 2e).^21^ Similar to our observations in the intestine, *S. maltophilia* feeding induced LD expansion in the hypodermis. The maximum LD diameter reached 6 μm (Figure 2e, Figure 2f and Figure 2g). Finally, we compared LD morphology in embryos from parents that were fed either OP50 or *S. maltophilia* (Figure 2h-2i). The embryos from *S. maltophilia*-fed worms again showed enlarged LDs (Figure 2i, white arrows). These results were further confirmed by examining isolated LDs from worms fed OP50 or *S. maltophilia*. The LDs isolated from *S. maltophilia-*fed larval L4 worms, were enlarged relative to OP50-fed worms (Figure 2j, Figure 2l, Figure 2k and Figure 2m). Our results indicate that dietary *S. maltophilia* caused LD enlargement in multiple tissues in worms.

The primary cargoes of LDs are neutral lipids, such as TAG. To determine if LD expansion observed in *S. maltophilia*-fed worms reflected an increase in organismal lipid content, we used biochemical methods to measure TAG levels. Total lipids were extracted from worms fed either OP50 or *S. maltophilia* from L1 to L4 stage. We found that *S. maltophilia*-fed worms had 3.7-fold more TAG than those fed OP50 (Figure 2n). Taken together, these morphological and biochemical analyses indicate that *S. maltophilia* can potently induce neutral lipid accumulation in *C. elegans*.

### *Induction of* C.elegans *neutral lipid storage by* S.maltophilia *was not due to its; species-specific fatty acid composition*

Differences in lipid composition between bacterial food sources could potentially underlie the effect of dietary *S. maltophilia* on host lipids, perhaps due to some lipids being more readily absorbed, processed, and incorporated than others. We examined the fatty acid compositions of *E. coli* OP50 and *S. maltophilia* by gas chromatography-mass spectrometry (GC-MS), and found substantial differences between them (Fig. S1A), and branched fatty acid iso 17(C17iso) was used to feed worm but no enlargement of LDs in the nematode intestinal cells was detected (Fig. S1F). However, it is not clear from this observation alone if lipid compositional difference accounted for the ability of *S. maltophilia* to promote neutral lipid accumulation in worms. Therefore, we conducted a transposon-based forward genetic screen in *S. maltophilia* to identify mutants that failed to induce LD expansion when fed to *C. elegans* (Fig. S1B). Through this approach, we isolated SMa9, a mutant *S. maltophilia* strain that could not increase LD size and number in *C. elegans* (Fig. S1C and S1D). However, there was no difference in fatty acid composition between the mutant SMa9 and the parental wild type (WT) bacterium (Fig. S1E). In addition, to detect the absorption rate of food fatty acids by nematodes, we fed worm with BODIPY 556/568 C12 as a free fatty acid in the presence OP50 or *S. maltophilia*, and the result showed that *S. maltophilia* did not affect uptake and incorporation of BODIPY 556/568 C12 (Fig. S8). Therefore, the results clearly suggest that one or more factors other than fatty acids, were responsible for the ability of *S. maltophilia* to promote neutral lipid storage in *C. elegans*.

### S. maltophilia-induced neutral lipid storage was not part of an innate immune response in C. elegans

We then examined whether the phenotype of excess neutral lipid accumulation was due to a pathological process associated with live *S. maltophilia*. Worms were fed ultraviolet (UV)-killed, culture supernatant of *S. maltophilia* or high temperature-killed *S. maltophilia* and their LDs were examined (Fig. S2A and S6). The killed bacteria retained the ability to increase LD size in *C. elegans*, compared with OP50 feeding (Fig. S2A). As the effect of the bacterial culture supernatant was very small, we concentrated the bacterial culture supernatant using freeze-drying method and fed the nematodes, and found that the bacterial culture supernatant could only induce the formation of nematode large LDs slightly (Fig. S6). Next, we measured the expression level of NLP-29,^34^ which is the nematode immune response marker. We found that NLP-29 could be significantly increased by *S. maltophilia* feeding (Fig. S2B). However, when we measured LD size in the worm defective in the p38 MAPK pathway (*sek-1, pmk-1*) that is critical for the innate immune response, we found no significant change in the LD phenotype in these mutant worms (Fig. S2C). We concluded that the elevated neutral lipid storage in worms upon *S. maltophilia* feeding was independent of the p38 MAPK pathway.

### S. maltophilia modulates lipid metabolism in C. elegans by upregulating sbp-1, fat-6,and fat-7

To understand the pathways that *S. maltophilia* utilize to enlarge LDs in the nematode, we explored other possible mechanisms of this process. To determine the effects of bacteria on *C. elegans* physiology, we measured developmental rate, fecundity, and hatching rate of worms fed by either *E. coli* OP50 or by *S. maltophilia*. To assess developmental rate, we synchronized animals at the L1 stage and monitored the developmental age of the population over time (Fig. S3A). Development to the L4 stage was delayed in worms fed by *S. maltophilia*, compared with OP50 (Fig. S3A). The number of offspring produced by *S. maltophilia*-fed worms was reduced by 30% compared with the OP50 group (Fig. S3B). There was no significant difference in hatching rate between OP50- and *S. maltophilia*-fed worms (Fig. S3C). The *S. maltophilia* bacteria were strongly preferred by worms over the *E. coli* OP50 (Fig. S3D-F). We measured pharyngeal pumping in worms after 1 h and 3 h of feeding and found no differences (Fig. S3G). Lastly, we investigated the effect of *S. maltophilia* feeding on LD size in *eat-4*(*ky5*) mutant worms, which exhibited reduced pharyngeal pumping and therefore ate less than wild type worms.^35^ There were no differences in LD size between wild type and *eat-4*(*ky5*) mutant worms when they were fed *S. maltophilia* (Fig. S3H). These results suggest that the effect of *S. maltophilia* on *C. elegans* LD size was not dependent on food intake.

**Figure 3.**
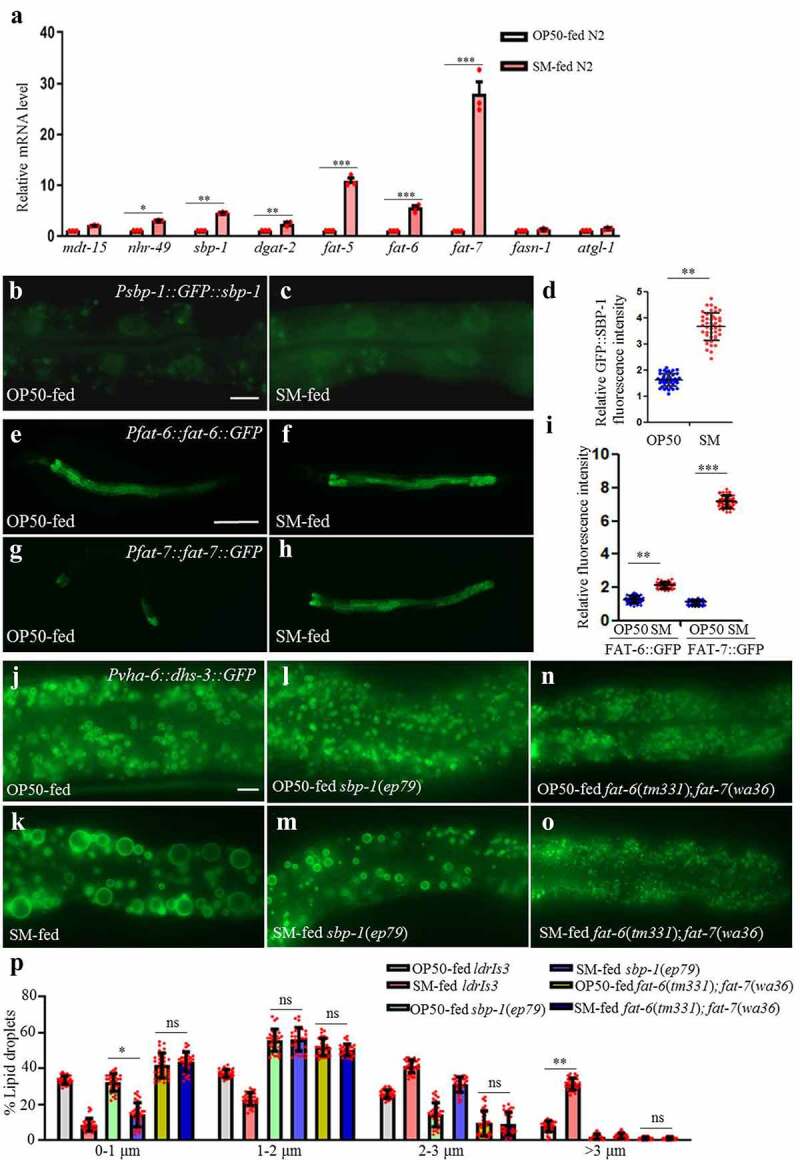
***S. maltophilia* induces the LD enlargement in *C. elegans* through *sbp-1, fat-6*, and *fat-7***. (a) RT-PCR result of the expression of lipid metabolism-related genes in OP50- and *S. maltophilia-*fed N2 worms. Data represent mean ± SEM (n = 3 for each independent experiment, student *t*-test, *P < .05, **P < .01, ***P < .001). (b) Fluorescence micrographs of GFP::SBP-1 in OP50-fed worms. Scale bar = 5 μm. (c) Fluorescence micrographs of GFP::SBP-1 in *S. maltophilia-*fed worms. (d) Quantification of the GFP::SBP-1 fluorescence intensity in b and c. Data are presented as mean ± SD of 45 animals for each worm strain, student *t*-test, **P < .01, n = 45. (e) Fluorescence micrographs of FAT-6::GFP in OP50-fed worms. Scale bar = 100 μm. (f) Fluorescence micrographs of FAT-6::GFP in *S. maltophilia-*fed worms. (g) Fluorescence micrographs of FAT-7::GFP in OP50-fed worms. (h) Fluorescence micrographs of FAT-7::GFP in *S. maltophilia-*fed worms. (i) Quantification of the fluorescence intensity in e, f, g, and h. Data are presented as mean ± SD of 45 animals for each worm strain, student *t*-test, **P < .01, ***P < .001, n = 45. (j) Fluorescence micrographs of *Pvha-6::dhs-3::GFP* labeled LDs in the intestine of OP50-fed worms. Scale bar = 5 μm. (k) Fluorescence micrographs of *Pvha-6::dhs-3::GFP* labeled LDs in *S. maltophilia-*fed worms. (l) Fluorescence micrographs of *Pvha-6::dhs-3::GFP* labeled LDs in *sbp-1*(*ep79*) mutant animals fed OP50. (m) Fluorescence micrographs of *Pvha-6::dhs-3::GFP* in *sbp-1*(*ep79*) mutant animals fed *S. maltophilia*. (n) Fluorescence micrographs of *Pvha-6::dhs-3::GFP* labeled LDs in *fat-6*(*tm331); fat-7*(*wa36*) double mutant animals fed by OP50. (o) Fluorescence micrographs of *Pvha-6::dhs-3::GFP* labeled LDs in *fat-6*(*tm331); fat-7*(*wa36*) double mutant animals fed by *S. maltophilia*. (p) Distribution of the LD size (%lipid droplets), as measured from *ldrIs3, sbp-1*(*ep79*) and *fat-6*(*tm331); fat-7*(*wa36*) double mutant animals fed by OP50 and by *S. maltophilia* from (j-o). Data are presented as mean ± SD of 30 animals for each worm strain, student’s *t*-test and one-way ANOVA, **P < .01, 0.01<*P < .05, ns, no significance, n = 30.

Next, we turned our attention to examine lipid metabolic pathways in *C. elegans*. The enlargement of LDs or increasing of TAG accumulation mainly depends on the increasing of TAG synthesis or/and decreasing of TAG hydrolysis.^36,37^ Therefore, we compared the levels of mRNA expression of TAG metabolism-related genes^38–41^ in OP50 *vs. S. maltophilia* fed worms, and the results are presented in Figure 3a. Among them the genes involved in synthesis of fatty acids especially unsaturated fatty acids were significantly upregulated. Previous studies also found that feeding fatty acids especially unsaturated fatty acids can increase LD size and TAG content.^42,43^ Wild type worms were fed OP50 or *S. maltophilia* from L1 to L4 stage and the relative expression level of selected metabolic genes was determined by real-time PCR. This analysis clearly indicated that dietary *S. maltophilia* significantly increased the expression of *sbp-1, fat-5, fat-6*, and *fat-7*, all of which are critical for lipogenesis in *C. elegans*, but there is no significant change in lipid synthesis key genes (*mdt-15, nhr-49, dgat-2, fasn-1*) and lipid hydrolysis key genes (*atgl-1*) (Figure 3a).

SBP-1 is a basic helix-loop-helix (bHLH) transcription factor homologous to the mammalian sterol regulatory element binding proteins (SREBPs) that is required for lipid synthesis.^42^ FAT-6 and FAT-7 are acyl-CoA desaturases that function downstream of SBP-1.^41,43–47^ To verify the influence of *S. maltophilia* on these pathways, we fed *S. maltophilia* to *Psbp-1::GFP::sbp-1, Pfat-6::fat-6::GFP*, and *Pfat-7::fat-7::GFP* transgenic animals.^48^ Feeding GFP::SBP-1 worms by *S. maltophilia* resulted in robust induction of fluorescence in the whole worm (Figure 3b, Figure 3c and Figure 3d). The expression of FAT-6::GFP and FAT-7::GFP was also increased with *S. maltophilia* feeding, compared to OP50 control (Figure 3e, Figure 3f, Figure 3g, Figure 3h and Figure 3i). We next determined the effect of *S. maltophilia* on *sbp-1*(*ep79*) mutant worms and *fat-6*(*tm331); fat-7*(*wa36*) double mutant worms. The ability of dietary *S. maltophilia* to increase LD size was blunted in *sbp-1*(*ep79*) animals (Figure 3j, Figure 3k, Figure 3l, Figure 3m, and Figure 3p). Furthermore, dietary *S. maltophilia* had no effect on *fat-6*(*tm331); fat-7*(*wa36*) double mutant worms (Figure 3j, Figure 3k, Figure 3n, Figure 3o, and Figure 3p). Taken together, these results suggest that the effect of *S. maltophilia* on host LDs required a function SBP-1/FAT-6/FAT-7 pathway.

### dpy-9 and acs-13 suppress the S. maltophilia-induced lipid droplet expansion

**Figure 4.**
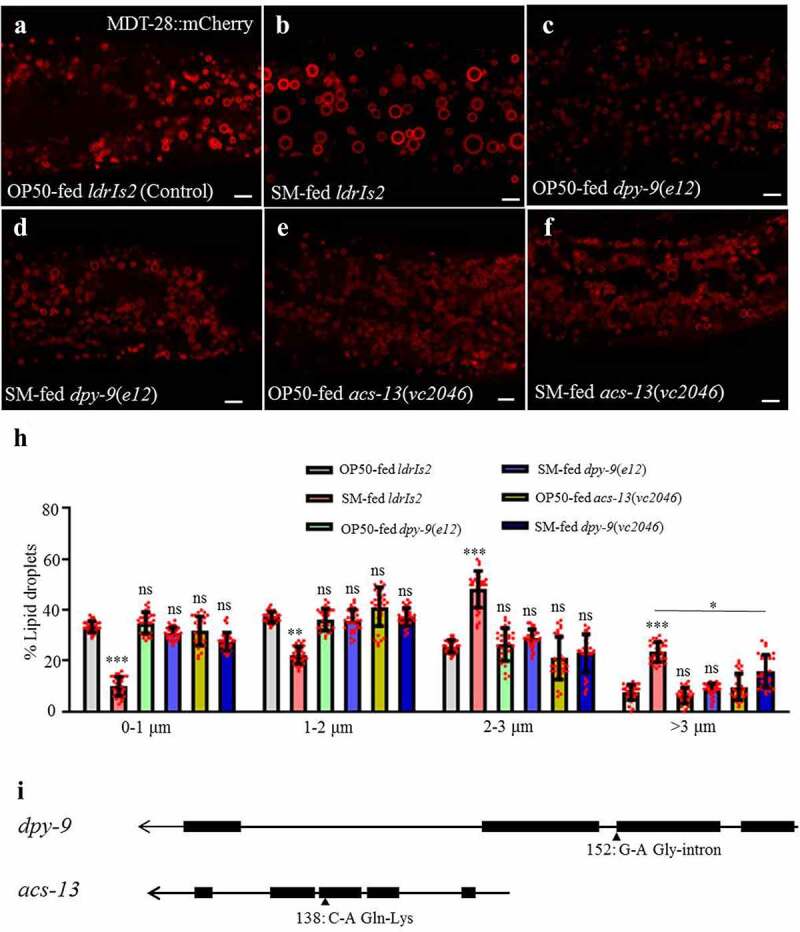
***dpy-9* and *acs-13* suppress the *S. maltophilia*-induced enlargement of LDs**. (a) Fluorescence micrographs of *Pmdt-28::mdt-28::mCherry* labeled LDs in OP50-fed worms. (b) Fluorescence micrographs of *Pmdt-28::mdt-28::mCherry* labeled LDs in *S. maltophilia-*fed worms. (c) Fluorescence micrographs of *Pmdt-28::mdt-28::mCherry* labeled LDs in *dpy-9*(*e12*) mutant animals fed by OP50. (d) Fluorescence micrographs of *Pmdt-28::mdt-28::mCherry* labeled LDs in *dpy-9*(*e12*) mutant animals fed by *S. maltophilia*. (e) Fluorescence micrographs of *Pmdt-28::mdt-28::mCherry* labeled LDs in *acs-13*(*vc2046*) mutant animals fed by OP50. (f) Fluorescence micrographs of *Pmdt-28::mdt-28::mCherry* labeled LDs in *acs-13*(*vc2046*) mutant animals fed by *S. maltophilia*. Scale bar = 5 μm. (g) Distribution of the LD size (% lipid droplets), as measured from (a-f). Data are presented as mean ± SD of 30 animals for each worm strain, student’s *t*-test and one-way ANOVA, 0.01<*P < .05, *** *P* < .001, ns, no significance, n = 30. (h) Schematic representation of the gene structures and mutation sites of *dpy-9* and *acs-13.*

A forward genetic screen was conducted to identify *C. elegans* host factors that mediate the *S. maltophilia* effect on LDs. We used DHS-3::GFP or MDT-28::mCherry as LD markers (Fig. 4a and 4b) and screened 7,000 haploid genomes after chemical mutagenesis with ethyl-methane sulfonate (EMS), and isolated 7 mutant strains (Figure 4a). Genetic mapping based on single nucleotide polymorphisms (SNPs) eventually led to the molecular cloning of two genes: *acs-13* and *dpy-9* (Figure 4c-Figure 4h). The *acs-13* gene encodes an ortholog of human ACSL1, 5, 6 (acyl-CoA synthetase long-chain family member 1, 5, 6). The *dpy-9* gene encodes a cuticular collagen family member with similarity to human collagen alpha 5, type IV. Using complementation tests and RNAi, we confirmed that the loss of *acs-13* and *dpy-9* function blocked the ability of *S. maltophilia* to induce LD expansion in *C. elegans* (Figure 4a–Figure 4i).

We next investigated if the loss of *acs-13* or *dpy-9* function could normalize neutral lipid storage in *C. elegans* mutants that are known to accumulate excess fat. Notably, mutations in *dhs-28* and *daf-22* are known to impair peroxisomal β-oxidation and induce LD expansion in the intestine in *C. elegans*.^49,50^ We analyzed OP50- and *S. maltophilia*-fed worms and found no significant difference in peroxisome morphology (Fig. S4A and S4B). Feeding *daf-22*(*m130*) mutants with *S. maltophilia* resulted in a significant increase in the number and size of LDs in the intestine and hypodermis (Fig. S4C and S4D), suggesting that dietary *S. maltophilia* acted in parallel of the peroxisomal β-oxidation pathway, which is responsible for fat catabolism. Finally, we recapitulated the LD expansion phenotype by knocking down *dhs-28* by RNAi in wild type worms, the enlarged LDs were detected in the intestine (Fig. S4E and S4F). Next, we knocked down *dhs-28* or *daf-22* in *acs-13* and *dpy-9* mutant worms. The *acs-13* mutation suppressed the formation of enlarged LDs in *dhs-28* or *daf-22* deficient worms. In contrast, mutations in *dpy-9* had no effect (Fig. S4G). Therefore, we concluded that DPY-9 is a host factor that is required for dietary *S. maltophilia* to promote LD expansion.

### S. maltophilia induces lipid droplet expansion by enhancing ER-LD interaction

The endoplasmic reticulum (ER) is the primary site where TAG is synthesized.^51,52^ Therefore, the effects of *S. maltophilia* feeding on the ER were investigated. We used two reporters, *Phyp-7::TRAM-1::GFP* (Fig. S5A and S5B) and *Pvha-6::SEL-1(1–79)::mCherry::HDEL*, to mark the ER membrane and lumen, respectively. After 2 days of *S. maltophilia* feeding, ring-shaped structures were observed in the hypodermis of the TRAM-1::GFP worms (Fig. S5C-5F). We also observed ER-wrapped LDs in the intestine of the mCherry::HDEL worms (Fig. S5G-5L). It is plausible that the remodeling of the ER morphology upon *S. maltophilia* feeding supports LD expansion.

**Figure 5.**
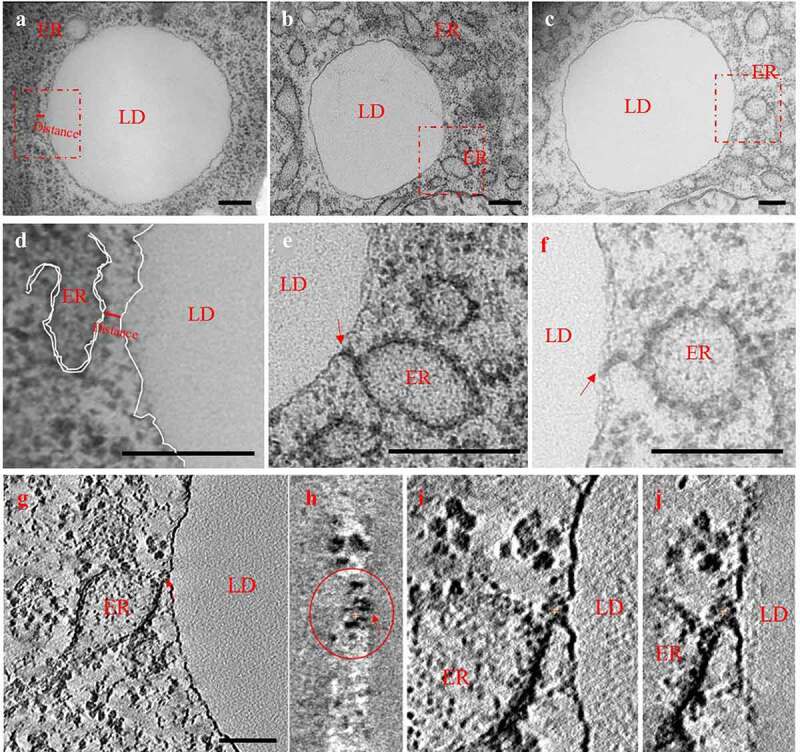
***S. maltophilia* induces the formation of ER-LD bridge**. (a) Morphology of ER and LD in N2 animals fed OP50. Scale bar = 200 nm. (b) Morphology of ER and LD in N2 fed by *S. maltophilia*. Scale bar = 200 nm. (c) As in (b), the other results for the morphology of ER and LD in N2 fed *S. maltophilia*. Scale bar = 200 nm. **(d, e, and f)** Insets enlarged for a, b, and c. Scale bar = 200 nm. The white dotted line depicts a single layer of phospholipid. (g) The ER-LD bridge in N2 fed *S. maltophilia* displayed using the tomography method. Scale bar = 100 nm. (h) Inset enlarged for G. (i and j) A different consecutive section and its enlarged inset.

We further investigated the ER-LD interaction using electron microscopy (EM). In worms fed by *E. coli* OP50 diet, few connections between the ER and LDs were observed (Figure 5a and Figure 5d), and there is no obvious structure between LD and ER. In contrast, in worms fed by *S. maltophilia* diet, we readily noted that LDs were connected to the ER through small tubular structures (Figure 5b, Figure 5c, Figure 5e, and Figure 5f). To observe the contact sites in detail, electron tomography was performed. The images show that the connection between LDs and the ER was approximately hollow (Figure 5g-Figure 5j). Since similar structures have previously been predicted by William A Prinz,^53^ we continue to name this structure ER-LD bridge.

To further understand how *S. maltophilia*-induced LD expansion was instigated by ER remodeling, we examined the localization of SEIP-1 that is highly enriched in an ER subdomain that associates tightly with LDs.^54^ SEIP-1 is the *C. elegans* ortholog of seipin that functions at ER-LD contact sites.^55–60^ To visualize SEIP-1 positive ER subdomain (peri-LD cages), we used two reporter strains that expressed SEIP-1::GFP fusion proteins either ubiquitously (*hjSi189*) or specifically in the intestine (*hjSi3*).^54^

**Figure 6.**
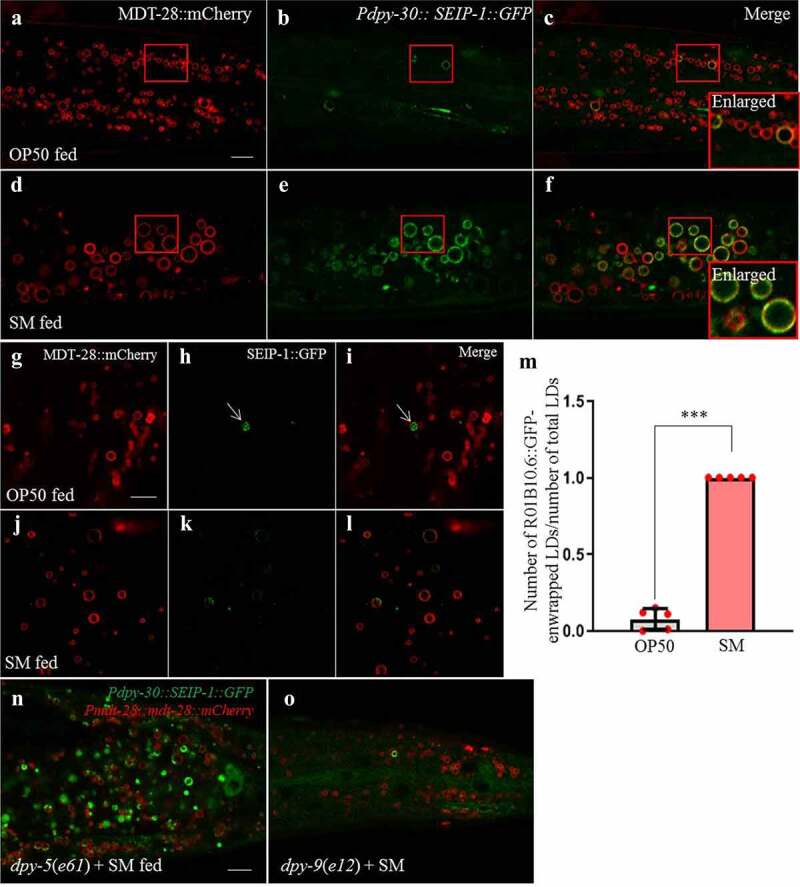
***S. maltophilia* induces SEIP-1::GFP-labeled ER enwrapping LDs**. (a) Fluorescence micrographs of *Pmdt-28::mdt-28::mCherry* labeled LDs in OP50-fed worms. Scale bar = 5 μm. (b) Fluorescence micrographs of *Pdpy-30::seip-1::gfp* labeled ER in OP50-fed worms. (c) As in (a), but with (b) merged. The red area is the enlarged position. (d) Fluorescence micrographs of *Pmdt-28::mdt-28::mCherry* labeled LDs in *S. maltophilia*-fed worms. (e) Fluorescence micrographs of *Pdpy-30::seip-1::gfp* labeled ER in *S. maltophilia*-fed worms. (f) As in (d), but with (e) merged. The red area is the enlarged position. (g) Fluorescence micrographs of LDs isolated from transgenic animal expressing MDT-28::mCherry. Scale bar = 5 μm. (h) Fluorescence micrographs of LDs isolated from OP50-fed transgenic animal expressing *Pdpy-30::seip-1::gfp*. (i) as in (g), but with GFP and mCherry signals merged. (j) Fluorescence micrographs of LDs isolated from *S. maltophilia*-fed transgenic animal expressing MDT-28::mCherry. (k) Fluorescence micrographs of LDs isolated from *S. maltophilia*-fed transgenic animal expressing *Pdpy-30::seip-1::gfp*. () as in (j), but with GFP and mCherry signals merged. (m) Quantification of ratio of the number of SEIP-1::GFP positive LDs to number of total LDs. Data represent mean ± SEM (n = 5 for each independent experiment, ***P < .001, student *t*-test). (n) Fluorescence micrographs of *Pmdt-28::mdt-28::mCherry* and *Pdpy-30::seip-1::gfp* in *dpy-5*(*e61*) mutant animals. Scale bar = 5 μm. (o) Fluorescence micrographs of *Pmdt-28::mdt-28::mCherry* and *Pdpy-30::seip-1::gfp* in *dpy-9*(*e12*) mutant animals.

When *hjSi189; ldrIs2* worms were fed *E. coli* OP50, few intestinal LDs were associated with SEIP-1::GFP cages (Figure 6a, Figure 6b and Figure 6c). In contrast, almost all intestinal LDs were enwrapped by SEIP-1::GFP cages when *hjSi189; ldrIs2* transgenic worms were fed *S. maltophilia*, coincident of LD expansion (Figure 6d, Figure 6e and Figure 6f). We also found that more than 95% of LDs were associated with SEIP-1::GFP positive structures in worms on *S. maltophilia* diet (Figure 6d, Figure 6e, and Figure 6f). To further quantified the fraction of LDs that were decorated with SEIP-1::GFP, LDs were isolated from *hjSi189; ldrIs2* worms fed by OP50 or by *S. maltophilia* for 2.5 days and analyzed using confocal microscopy. Again, few LDs were found to retain SEIP-1::GFP in the OP50-fed group while 100% of LDs from the *S. maltophilia*-fed group showed SEIP-1::GFP signals (The green SEIP-1::GFP signals appears more or less on the red MDT-28:: mCherry signal) (Figure 6g-Figure 6m). These results are consistent with the model that the SEIP-1 positive ER subdomain promotes LD expansion, and that *S. maltophilia* feeding significantly increases the coverage of such subdomain, thereby supporting widespread LD expansion.

Next, we investigated if loss of DPY-9 function could suppress *S. maltophilia*-induced LD expansion by interfering with SEIP-1 enrichment in peri-LD cages. Accordingly, we introduced *dpy-9*, and *dyp-5* (negative control) mutations into the SEIP-1::GFP reporter strain *hjSi189*. After feeding *S. maltophilia* for 2 days, no SEIP-1::GFP positive, peri-LD cages were observed in *dpy-9* mutant animals (Figure 6n, and Figure 6o). It is conceivable that DPY-9 directly or indirectly act in pathways that remodel the membrane environment of peri-LD cages. Such remodeling is crucial for the recruitment of SEIP-1 and additional proteins that are critical for LD expansion.

***Requirement of proteins involved in ER-LD*** ***interactions for S. maltophilia***-***induced*** ***lipid droplet expansion***

**Figure 7.**
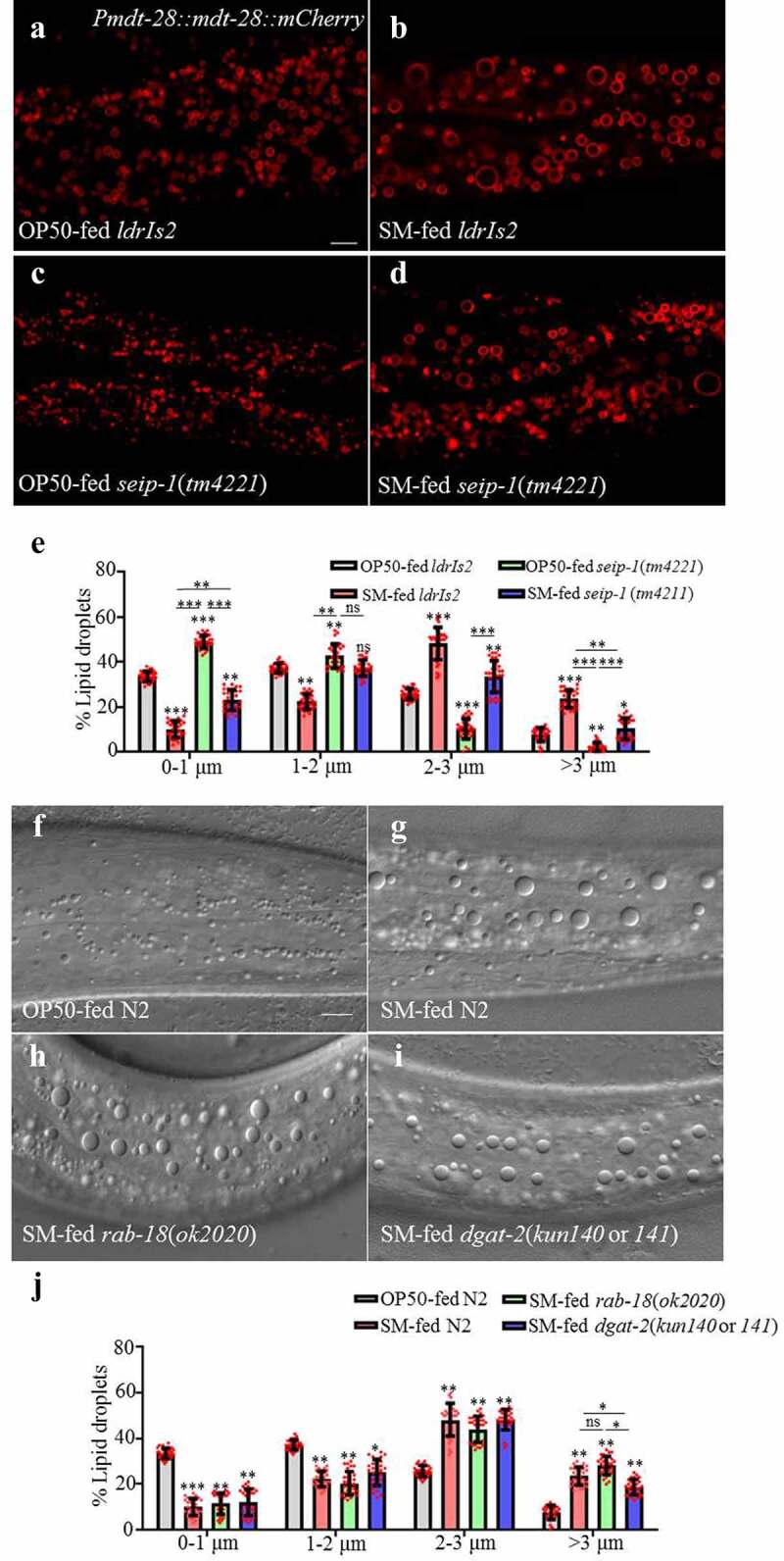
**Proteins involved in ER-LD interaction partially suppress the *S. maltophilia-*enlarged LDs**. (a) Fluorescence micrographs of *Pmdt-28::mdt-28::mCherry* labeled LDs in OP50-fed worms. Scale bar = 5 μm. (b) Fluorescence micrographs of *Pmdt-28::mdt-28::mCherry* labeled LDs in *S. maltophilia*-fed worms. (c) Fluorescence micrographs of *Pmdt-28::mdt-28::mCherry* labeled LDs in *seip-1*(*tm4221*) mutant animals fed by OP50. (d) Fluorescence micrographs of *Pmdt-28::mdt-28::mCherry* labeled LDs in *seip-1*(*tm4221*) mutant animals fed by *S. maltophilia*. (e) Distribution of the LD size (% lipid droplets) for (a-d). Data are presented as mean ± SD of 30 animals for each worm strain, student’s *t*-test and one-way ANOVA, ns, no significance, 0.01<*P < .05, *** *P* < .01, *** *P* < .001, n = 30. (f) DIC images of LDs in OP50-fed worms. Scale bar = 5 μm. (g) DIC images of LDs in worms fed *S. maltophilia*. (h) DIC images of LDs in *rab-18*(*ok2020*) mutant animals fed *S. maltophilia*. (i) DIC images of LDs in *dgat-2*(*kun140* or *141*) mutant animals fed by *S. maltophilia*. (j) Distribution of the LD size (% lipid droplets) for (f-i). Data are presented as mean ± SD of 30 animals for each worm strain, student’s *t*-test and one-way ANOVA, 0.01<*P < .05, *** *P* < .01, *** *P* < .001, ns, no significance, n = 30.

To determine if SEIP-1 is important for LD expansion induced by dietary *S. maltophilia*, we fed *S. maltophilia* to *seip-1*(*tm4221*) mutant animals (Figure 7a, Figure 7b, Figure 7c, and Figure 7d). The results revealed that loss of SEIP-1 function only partially suppressed the effect of *S. maltophilia* feeding on LDs (Figure 7e), which hints that additional proteins may reside in ER-LD contacts that mediate the effect of dietary *S. maltophilia*. To this end, we investigated *rab-18* and *dgat-2* since they have been reported to be involved in ER-LD interaction.^61–65^ We examined the effect of *rab-18*, and *dgat-2* mutations on the size of LDs in *C. elegans* after *S. maltophilia* feeding. As shown in Figure 7f-Figure 7j, loss of DGAT-2 function partially suppressed LD expansion. However, loss of Rab-18 function had no effect in this experimental context.

## Discussion

**Figure 8.**
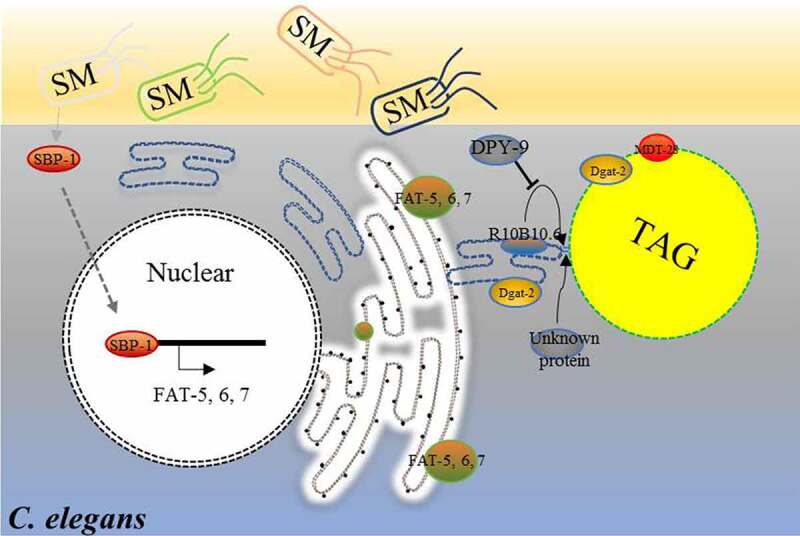
**Model for the enlargement of LDs in *C. elegans* fed by *S. maltophilia.*** Different bacteria have different effects on the obesity of nematodes. Compared with the standard *E. coli* OP50, *S. maltophilia* induces the accumulation of enlarged LDs through an increase in lipogenesis by upregulating genes (*sbp-1*/*fat-6; fat-7*), and increasing the interaction between ER and LDs in nematodes. The ER-LD bridge likely facilitates the transfer of lipid from ER to LDs in *C. elegans.*

In this paper, we show that dietary *S. maltophilia* promoted organismal neutral lipid storage in *C. elegans* through a lipogenic transcriptional program that consists of SBP-1, FAT-6 and FAT-7. In addition, *S. maltophilia* feeding enhanced DPY-9-dependent ER-LD interaction in the intestine and hypodermis, which was partially dependent on DPY-9. Collectively, we hypothesize that the increased contact, via ER-LD bridges, facilitates the transfer of lipid from the ER to LDs (Figure 8).

To investigate the physiological significance of *S. maltophilia*-enlarged LDs in *C. elegans*, we conducted experiments to determine if they affect survival. The lifespan of worms fed *S. maltophilia* was not significantly changed compared with worms fed OP50 (Fig. S7). However, blocking the *S. maltophilia*-induced increase in LD size by knocking out *sbp-1* or *fat-6; fat-7* reduced the lifespan of the worms (Fig. S7B and S7C). Next, we fed N2 worms *S. maltophilia* for 2 days to induce large LDs. Subsequently the animals were infected with PA14 (Fig. S7D). *S. maltophilia*-fed worms were able to resist PA14 infection better than the OP50-fed worms (Fig. S7E). This indicates that the emergence of enlarged LDs may protect nematodes from environmental stress, which is important for their survival.

*C. elegans* and its diet has become a powerful system to study host-microbiota interactions.^5,9,18^ However, it is still challenging to study the relationship between bacteria and lipid metabolism in nematodes because bacteria not only provide nutrients, but also can be pathogenic to the worms.^5^ The observation that UV-killed *S. maltophilia* still generated the LD phenotype suggests that the effect is not due to pathogenicity. To examine the role of bacterial lipids in the phenotype, gas chromatography-mass spectroscopy was used to measure the fatty acid composition in *S. maltophilia* and OP50. The fatty acids of *S. maltophilia* are mainly 13-methyl myristic acid (C15iso) and 15-methyl hexadecanoic acid (C17iso), which differs substantially from those of OP50. We investigated further using a transposon-based forward genetic screen in *S. maltophilia* and found that a mutation in SMa9 lost the effect in *C. elegans*. We compared the fatty acid composition of *S. maltophilia* and SMa9 and found that their fatty acids were not significantly different, which rules out a causal role of fatty acid composition.

The use of *C. elegans* to study host-pathogen interaction was pioneered by the Ausubel lab, which stemmed from the observations that *Pseudomonas Aeruginosa* PA14 could effectively kill worms.^66^ *C. elegans* genetic and molecular analyses led to the discovery of conserved innate immune response pathways that counter *P. Aeruginosa* infection.^67,68^ The systematic response appears to require environmental sensing and inter-tissue communication between the nervous system, hypodermis and intestine.^69–71^ Ultimately, multiple transcriptional networks coordinately mount gene expression programs that support the defense against the pathogen.^72,73^ Besides *P. Aeruginosa*, many other pathogenic bacteria, such as *E. faecalis, S. aureus, S. typhimurium* and *C. neoformans*, have subsequently been shown to effectively kill worms.^74^ However, relatively less is known about the impact of pathogenic bacteria on *C. elegans* physiology during chronic infection. Here, we discovered that environmental isolates of the pathogenic bacterium *S. maltophilia* did not kill worms, and may be able to colonize the intestines.^28^ Instead, worms maintained on a *S. maltophilia* diet grew slower and accumulated excess neutral lipid, but for *unc-76* or *daf-2* mutants, the slow-growing mutant, *S. maltophilia* still had the ability to induce LD expansion (data unpublished), this excluded the effect of growth on the enlargement of LDs. Accordingly, the p38 MAP kinase pathway, which is central to innate immunity against lethal pathogens in *C. elegans*, did not play a major role in the induction of neutral lipid storage in response to dietary *S. maltophilia*. Our results indicate that *S. maltophilia* may exert metabolic burden to its host. Further investigation is needed to elucidate the casual factors from *S. maltophilia* that modulate host metabolism.

Since *S. maltophilia* is commonly found in immunocompromised, hospitalized patients,^75^ there is an urgent need to understand the host response to this opportunistic pathogen, in order to identify cellular proteins that may serve as therapeutic targets. From a forward genetic screen, we found that loss of function mutations in *dpy-9* rendered the worms resistant to *S. maltophilia*-induced neutral lipid accumulation. The *dpy-9* gene encodes an extracellular matrix collagen that is presumably expressed in the hypodermis. The lack of *dpy-9* compromises cuticle integrity that may give rise to osmotic stress.^76^ Based on the functional annotation of this protein, it is currently unclear how the loss of DPY-9 function in distinct tissues confers resistance to *S. maltophilia*. To further understand its function, we further examined the epidermal structure of the nematode and found that *S. maltophilia* feeding only increased the fluorescence intensity of COL-19::GFP, but it could not change its texture (Fig. S10). It is plausible that DPY-9 is part of a wider genetic network that facilitates inter-tissue communication in response to *S. maltophilia*. Phosphatidylcholine (PC) and phosphatidylethanolamine (PE) are the most abundant phospholipids in cell membranes and the relative abundance of PC and PE regulates the size and dynamics of LDs.^54,77,78^ Based on our results, the ratio of PC to PE was not the reason for *S. maltophilia*-induced large LDs. Although further analysis is needed to identify other components of this network, our results clearly suggest that one of its outputs is required to sustain ER-LD contacts, which in turn supports neutral lipid accumulation in LDs.

Our work provides a basis for further investigation into the mechanisms governing ER-LD interaction. Under normal conditions, it is difficult to identify and study proteins involved in this process, because ER-LD contact is rare and not easily detected. However, the ER-LD interactions induced by *S. maltophilia* provide an unambiguous, robust, and easily measured phenotype dependent on the machinery of this inter-organelle contact. This discovery sets the stage for further investigation using genetic screening and mass spectrometry to further elucidate the mechanistic basis for this phenomenon.

To further investigate the role of enlarged LDs in *S. maltophilia*-fed worms, we examined the percent survival of the nematodes. This experiment showed that the emergence of highly enlarged LDs enhanced the resistance of nematodes to stress. Based on this finding, we hypothesize that LDs may sequester or eliminate some toxic substances, giving the animals a survival advantage in extreme conditions.

In summary, our work has established a new host-microbe experimental paradigm. Chronic exposure of *C. elegans* to *S. maltophilia* reproducibly caused metabolic remodeling in multiple tissues. Future experiments will be directed to studying microbial factors that are responsible for such remodeling via DPY-9 and additional yet-to-be identified host factors.

## Conclusions

**1)** Bacterium *S. maltophilia* stimulates the formation of supersized LDs in *C. elegans*, **2)** The effect of *S. maltophilia* on LDs is mediated by the *sbp-1, fat-6*, and *fat-7* pathway, **3)** an ER-LD bridge is involved in the *S. maltophilia*-induced effect on LDs. These findings suggest that *C. elegans* can serve as a model organism for the study of gut microbiota-mediated human obesity.

## Materials and Methods

### Nematode strains and growth conditions

All *C. elegans* strains were handled and maintained following standard procedures.^8^ The N2 Bristol strain, *dpy-9*(*e12*), and *dpy-5*(*e61*) were obtained from the *Caenorhabditis* Genetics Center. *sbp-1*(*ep79), fat-6*(*tm331), fat-7*(*wa36), epEx307*[*Psbp-1::GFP::sbp-1*], [*Pfat-7::fat-7::GFP*], and [*Pfat-6::fat-6::GFP*] were gifts from Bin Liang’s laboratory. *seip-1*(*tm4211*) was obtained from Xun Huang’s laboratory. *hjSi158*[*vha-6p::SEL-1(1–79)::mCherry::HDEL::let-858 3ʹ UTR], hjSi3*[*vha-6p::seip-1 cDNA::GFP_TEV_3xFLAG::let-858 3ʹ UTR], ldrIs3*[*vha-6p::dhs-3::gfp*], and *hjSi189*[*dpy-30p::seip-1 cDNA::GFP_TEV_3xFLAG::tbb-2 3ʹ UTR*] were from Ho Yi Mak’s laboratory. *sek-1*(*km4), pmk-1*(*ku54), Phyp-7::tram-1::gfp*, and *Phyp-7::PS1::gfp* were from Hong Zhang’s laboratory. *ldrIs2*[*mdt-28p::mdt-28::mCherry, unc-76(+)*] strains were constructed in our laboratory.

*S. maltophilia* and OP50 were cultured on LB and NGM plates.

All experimental animals were maintained at 20°C.

### Forward genetic screen and mutant mapping

To screen the mutant animals with a suppressed phenotype of LD induced by *S. maltophilia, ldrIs3* was mutagenized with ethyl methane sulfonate (EMS) as previously described.^79,80^ 7,000 haploid genomes were screened and mutant phenotype in the F2 worms were selected to observe the LD phenotype. The mutant animals were selected with a suppressed phenotype of LD induced by *S. maltophilia* (Mutants which LDs could not become larger on a *S. maltophilia* diet). Mutant worms were backcrossed with *ldrIs3* at least four times and their LD phenotypes were studied using a ZEISS Imager M2. The stable mutant animals were mapped using single-nucleotide polymorphism (SNP) mapping.^81^

### Isolation of lipid droplets

LDs were isolated using a method described previously.^20,82^ Briefly, synchronized L1 worms were cultured on NGM plates and the L4 larval stage worms were collected in M9 buffer (22 mM KH_2_PO_4_, 42 mM Na_2_HPO_4_, 86 mM NaCl, 1 M MgSO_4_). The worms were washed three times in Buffer A (25 mM Tricine, pH 7.6, 250 mM Sucrose, and 0.2 mM Phenylmethylsulfonyl fluoride) and were then homogenized to obtain whole body lysates. The lysates were centrifuged at different speeds (2,020 *g*, 4,546 *g* and 12,628 *g*) in SW40 tubes to obtain LDs of different diameters. The LD fraction was collected from the top layer and was washed three times with Buffer B (20 mM HEPES, 100 mM KCl, 2 mM MgCl_2_, pH 7.4). Images of the isolated LDs were obtained using a ZEISS Imager M2 or LSM880.

### Measurement of triacylglycerol

Synchronized L4 worms were washed three times with M9, and were dissolved in 200 μl 1% Triton X-100 by sonication. Then, the worm lysates were centrifuged at 13,210 *g* for 3 min. The triacylglycerol (TAG) content of the supernatants was measured using the Triacylglycerol Assay Kit.^19^ The Pierce BCA Protein Assay Kit (Thermo, USA) was used to quantify the proteins.^19^ The results are shown in the ratio of TAG to protein (PRO) (mg/mg).

### Lipid extraction and analysis

Bacterial lipid extraction, separation, and analysis were conducted as described previously.^43^ In general, bacteria were grown at 20°C on the NGM plates, after 24 h, 3 ~ 5 mg bacteria were collected into glass tubes and water was removed with a Pasteur pipet. To each bacterial pellet 1 ml of MeOH and 2.5% H_2_SO_4_ was added. Fatty acids were extracted and converted into methyl esters by heating at 70°C for 60 min. Then the extractions were incubated at 25°C for 5 min followed by addition of 0.2 ml of hexane and 1.5 ml of water. The mixtures were shaken vigorously and then centrifuged 12,000 *g* for 1 min in a clinical centrifuge. The fatty acid methyl esters contained in the top hexane-rich fraction were collected and 2 μl were analyzed using an Agilent 6890 series gas chromatograph equipped with a 20 m x 0.25 mm SP-2380 column (Supelco, Bellefonte, PA) and a flame ionization detector. The Gas Chromatography (GC) was programmed for an initial temperature of 120°C for 1 min followed by an increase of 10°C per min up to 190°C followed by an increase of 2°C per min to 200°C. Peak identity was determined by mass spectroscopy.

### Transposon-based forward genetic screen

The *S. maltophilia* transposon insertion mutant library was constructed using an EZ::TN <KAN-2> Tnp transposome kit (Epicenter) as described previously and in accordance with the manufacturer’s protocol.^83^ The kanamycin-resistant transformants were selected and fed the worms. To identify mutants which did not result in enlarged LDs when fed to *C. elegans*.

### Fluorescence imaging of *C.elegans*

Worms were immobilized with 0.5 mg/ml levamisole in M9 buffer and then transferred to a small glass slide or dish, then covered by a 2–4% (w/v) agar pad. For GFP or LipidTOX (G), a 488 nm laser was used for excitation and signals were collected with a 500–550 nm emission filter. For mCherry, a 561 nm laser was used for excitation and signals were collected with a 585–650 nm emission filter. For auto-fluorescence in the intestine, a 405 nm laser was used for excitation and signals were collected with a 417–477 nm emission filter. To obtain optimal images, immersion oils with refractive indices of 1.520 were used for LD on glass coverslips. Fluorescence images of L4 larval animals were obtained using a laser scanning confocal microscope (LSM 710 Meta, LSM880 Meta, ZEISS). Fluorescence images were taken and used to measure the diameter of the LDs in the posterior of the intestine with the same area (100 μm × 80 μm) by Image J and ZEN 2011 (ZEISS). At least 10 worms were utilized to measure LD size for each worm strain. All LDs were manually counted in the images. The Image J software usage method is as follows: select “Analyze”-“Set Scale”-“Known distance input the length of the drawn straight line”-“Unit of length input unit, check the Global checkbox (this standard is used for all pictures)”-click “OK”-“Ctrl+M (Measurement), the result will be displayed and recorded in the Results window”-“Length displays the converted length value”, and the measurement method was done blinded. According to the diameter of the LDs, the LDs were divided into 0–1 μm, 1–2 μm, 2–3 μm, and >3 μm. % LDs represents Number of LDs with certain size/Total number of LDs. All experiments are repeated three times.

LD size measurements were made at the L4 larval stage because: (1) the L4 stage can be easily identified; (2) earlier larval stages are too small for imaging; and (3) at the adult stage, embryos will interfere with imaging and may affect LD morphology.

### Lifespan assay for *C.* *elegans*

Lifespan analysis was conducted at 20°C according to a protocol modified from a previous method.^84,85^ Briefly, synchronized L1 animals were seeded onto NGM plates and grown until the L4 stage. On day 0, 20 L4 worms per plate (five plates, about 100 worms in total per condition) were transferred onto different diets plates. The statistical analyses were performed using GraphPad Prism 5.

### Quantitative RT-PCR analysis

Total RNA was isolated using Trizol reagent.^86^ Moloney murine leukemia virus (M-MuLV) reverse transcriptase with random hexamer primers was used to synthesize the cDNA. RT-PCR was performed on a CFX96 real-time system with SYBR green. Relative expression levels of all mRNAs were normalized to *ama-1* mRNA.

### Behavioral experiment

OP50 and *S. maltophilia* were cultured and dropped on NGM plates. The bacteria were allowed to grow for 24 h. The synchronized L4 larval nematodes were placed equidistant between the OP50 and *S. maltophilia* cultures. After 12 h, the number of nematodes on the bacterial colonies was determined.

### RNAi assay of *C.elegans*

L4440 (HT115) was used as the control for the RNAi assay.^19^ The RNAi for *dhs-28* and *daf-22* were from the Ahringer RNAi library. The synchronized L1 (P0) worms were cultured on the RNAi NGM plates at 20°C to generate F1 or F2 worms for phenotypic analysis.

### Brood size analysis

Approximately 20 L4 worms were removed from synchronized mothers, fed with OP50, and transferred to NGM plates seeded with the appropriate bacteria, in triplicate. The worms were transferred to new plates every 24 h three times until no additional embryos were produced. The number of embryos was counted every 24 h.^9,87^

### Growth rate

Two-day old adults fed with the relevant bacteria were treated with hypochlorite to obtain embryos. The embryos were plated on NGM plates (∼100/plate) seeded with different bacteria. The percentage of L4 worms out of the total worms was determined at various time points.^19^

### Pharyngeal pumping measurements

L4 stage worms were picked from NGM plates. After 24 h, the pumping rate on different food was recorded. Worms were detected under an optical microscope and pumping rates were measured by visual observation. One pharyngeal pump was defined as a complete forward and backward movement of the grinder in the pharynx. The rate was counted for a period of 30 sec.^88^

### Dietary supplements

Fatty acid C17iso (From Prof. Huanhu Zhu) was prepared as a 10 mM stock in DMSO. A stock solution was mixed with 500 μl of OP50 overnight bacterial suspension in a 1:10 ratio and spotted on the appropriate plates according to the experiment.^89^

### Electron microscopy assay

Samples were prepared using previously described methods.^90^ 2D images were acquired using transmission electron microscope (FEI Tecnai Sprit 120 kV). 3D images were acquired using transmission electron microscope (FEI Tecnai Sprit 120 kV and Serial EM software).

### Data analysis

All numerical data were plotted as mean ± SEM unless otherwise indicated. The statistical analysis was performed using GraphPad Prism 8 and Image J (NIH, USA). Data represent biological replicates. Appropriate statistical tests were used for every figure. Data met the assumptions of the statistical tests described for each figure. Statistical parameters, including the exact value of n and descriptive statistics (mean ± SEM) and statistical significance, are reported in the figures and the figure legends. Data are judged to be statistically significant when *P* < .05 by two-tailed Student’s *t* test. In figures, asterisks denote statistical significance as calculated by Student’s *t* test (*P < .05, **P < .01, and ***P < .0001), as compared to appropriate controls.

## Supplementary Material

Supplemental Material

## Data Availability

All data generated or analyzed during this study are included in this published article and its supplementary information files.

## Abbreviations

*C. elegans, Caenorhabditis elegans*; LD, lipid droplet; ER, Endoplasmic reticulum; *S. maltophilia* (SM), *Stenotrophomonas maltophilia*; SMa9, *Stenotrophomonas maltophilia* mutant 9; SNP, Single-nucleotide polymorphism; TAG, triacylglycerol; PA14, Pseudomonas aeruginosa (strain UCBPP-PA14); NGM, Nematode Growth Medium; WT, wild type; EMS, Ethyl methane sulfonate; CGC, Caenorhabditis Genome Center; NBRP, National BioResource Project; ATCC, American Type Culture Collection.

## References

[1] Montalvo-Katz S, Huang H, Appel MD, Berg M, Shapira M, Adams JH. Association with soil bacteria enhances p38-dependent infection resistance in Caenorhabditis elegans. Infect Immun. 2013;81(2):514–22. doi:10.1128/IAI.00653-12. 23230286 PMC3553824

[2] Felix MA, Duveau F. Population dynamics and habitat sharing of natural populations of Caenorhabditis elegans and C. briggsae. BMC Biol. 2012;10(1):59. doi:10.1186/1741-7007-10-59. 22731941 PMC3414772

[3] Duveau F, Felix MA, Noor MAF. Role of pleiotropy in the evolution of a cryptic developmental variation in Caenorhabditis elegans. PLoS Biol. 2012;10(1):e1001230. doi:10.1371/journal.pbio.1001230. 22235190 PMC3250502

[4] Avery L, Shtonda BB. Food transport in the C. elegans pharynx. J Exp Biol. 2003;206(Pt 14):2441–2457. doi:10.1242/jeb.00433. 12796460 PMC3951750

[5] Zhang J, Holdorf AD, Walhout AJ. C. elegans and its bacterial diet as a model for systems-level understanding of host-microbiota interactions. Curr Opin Biotechnol. 2017;46:74–80. doi:10.1016/j.copbio.2017.01.008. 28189107 PMC5544573

[6] Watson E, MacNeil LT, Ritter AD, Yilmaz LS, Rosebrock AP, Caudy AA, Walhout AJM. Interspecies systems biology uncovers metabolites affecting c. elegans gene expression and life history traits. Cell. 2014;156(6):1336–1337. doi:10.1016/j.cell.2014.02.036. 28898637

[7] Sulston JE, Brenner S. The DNA of Caenorhabditis elegans. Genetics. 1974;77(1):95–104. doi:10.1093/genetics/77.1.95. 4858229 PMC1213121

[8] Brenner S. The genetics of Caenorhabditis elegans. Genetics. 1974;77(1):71–94. doi:10.1093/genetics/77.1.71. 4366476 PMC1213120

[9] Brooks KK, Liang B, Watts JL, Melov S. The influence of bacterial diet on fat storage in C. elegans. PLoS One. 2009;4(10):e7545. doi:10.1371/journal.pone.0007545. 19844570 PMC2760100

[10] Virk B, Correia G, Dixon DP, Feyst I, Jia J, Oberleitner N, Briggs Z, Hodge E, Edwards R, Ward J, *et al*. Excessive folate synthesis limits lifespan in the C. elegans: e. coli aging model. BMC Biol. 2012;10(1):67. doi:10.1186/1741-7007-10-67. 22849329 PMC3583181

[11] Scott TA, Quintaneiro LM, Norvaisas P, Lui PP, Wilson MP, Leung KY, Herrera-Dominguez L, Sudiwala S, Pessia A, Clayton PT, *et al*. Host-microbe co-metabolism dictates cancer drug efficacy in C. elegans. Cell. 2017;169(3):442–456 e418. doi:10.1016/j.cell.2017.03.040. 28431245 PMC5406385

[12] Garcia-Gonzalez AP, Ritter AD, Shrestha S, Andersen EC, Yilmaz LS, Walhout AJM. Bacterial metabolism affects the C. elegans response to cancer chemotherapeutics. Cell. 2017;169(3):431–441 e438. doi:10.1016/j.cell.2017.03.046. 28431244 PMC5484065

[13] MacNeil LT, Watson E, Arda HE, Zhu LJ, Walhout AJ. Diet-induced developmental acceleration independent of TOR and insulin in C. elegans. Cell. 2013;153(1):240–252. doi:10.1016/j.cell.2013.02.049. 23540701 PMC3821073

[14] Watson E, MacNeil LT, Ritter AD, Yilmaz LS, Rosebrock AP, Caudy AA, Walhout AJ. Interspecies systems biology uncovers metabolites affecting C. elegans gene expression and life history traits. Cell. 2014;156(4):759–770. doi:10.1016/j.cell.2014.01.047. 24529378 PMC4169190

[15] Gusarov I, Gautier L, Smolentseva O, Shamovsky I, Eremina S, Mironov A, Nudler E. Bacterial nitric oxide extends the lifespan of C. elegans. Cell. 2013;152(4):818–830. doi:10.1016/j.cell.2012.12.043. 23415229

[16] Qi B, Han M. Microbial siderophore enterobactin promotes mitochondrial iron uptake and development of the host via interaction with ATP synthase. Cell. 2018;175(2):571–582 e511. doi:10.1016/j.cell.2018.07.032. 30146159

[17] Han B, Sivaramakrishnan P, Lin CJ, Neve IAA, He J, Tay LWR, Sowa JN, Sizovs A, Du G, Wang J, *et al*. Microbial genetic composition tunes host longevity. Cell. 2017;169(7):1249–1262 e1213. doi:10.1016/j.cell.2017.05.036. 28622510 PMC5635830

[18] Lin CJ, Wang MC. Microbial metabolites regulate host lipid metabolism through NR5A-Hedgehog signalling. Nat Cell Biol. 2017;19(5):550–557. doi:10.1038/ncb3515. 28436966 PMC5635834

[19] Liu Y, Xu S, Zhang C, Zhu X, Hammad MA, Zhang X, Christian M, Zhang H, Liu P, Scott TA. Hydroxysteroid dehydrogenase family proteins on lipid droplets through bacteria, C. elegans, and mammals. Biochim Biophys Acta Mol Cell Biol Lipids. 2018;1863(8):881–894. doi:10.1016/j.bbalip.2018.04.018. 29702244

[20] Zhang P, Na H, Liu Z, Zhang S, Xue P, Chen Y, Pu J, Peng G, Huang X, Yang F, *et al*. Proteomic study and marker protein identification of Caenorhabditis elegans lipid droplets. Mol Cell Proteomics. 2012;11(8):317–328. doi:10.1074/mcp.M111.016345. 22493183 PMC3412964

[21] Na H, Zhang P, Chen Y, Zhu X, Liu Y, Liu Y, Xie K, Xu N, Yang F, Yu Y, *et al*. Identification of lipid droplet structure-like/resident proteins in Caenorhabditis elegans. Biochim Biophys Acta. 2015;1853(10):2481–2491. doi:10.1016/j.bbamcr.2015.05.020. 26025681

[22] Salans LB, Cushman SW, Weismann RE. Studies of human adipose tissue. Adipose cell size and number in nonobese and obese patients. J Clin Invest. 1973;52(4):929–941. doi:10.1172/JCI107258. 4693656 PMC302341

[23] Spalding KL, Arner E, Westermark PO, Bernard S, Buchholz BA, Bergmann O, Blomqvist L, Hoffstedt J, Naslund E, Britton T, *et al*. Dynamics of fat cell turnover in humans. Nature. 2008;453(7196):783–787. doi:10.1038/nature06902. 18454136

[24] Sahini N, Borlak J. Genomics of human fatty liver disease reveal mechanistically linked lipid droplet-associated gene regulations in bland steatosis and nonalcoholic steatohepatitis. Transl Res. 2016;177:41–69. doi:10.1016/j.trsl.2016.06.003. 27376874

[25] Seebacher F, Zeigerer A, Kory N, Krahmer N. Hepatic lipid droplet homeostasis and fatty liver disease. Semin Cell Dev Biol. 2020;108:72–81. doi:10.1016/j.semcdb.2020.04.011. 32444289

[26] Schulze RJ, Ding WX. Lipid droplet dynamics in alcoholic fatty liver disease. Liver Res. 2019;3(3–4):185–190. doi:10.1016/j.livres.2019.09.002. 33664985 PMC7928432

[27] White CV, Darby BJ, Breeden RJ, Herman MA, Payne SM. A stenotrophomonas maltophilia strain evades a major caenorhabditis elegans defense pathway. Infect Immun. 2016;84(2):524–536. doi:10.1128/IAI.00711-15. 26644380 PMC4730580

[28] Zimmermann J, Obeng N, Yang W, Pees B, Petersen C, Waschina S, Kissoyan KA, Aidley J, Hoeppner MP, Bunk B, *et al*. The functional repertoire contained within the native microbiota of the model nematode caenorhabditis elegans. ISME J. 2020;14(1):26–38. doi:10.1038/s41396-019-0504-y. 31484996 PMC6908608

[29] Radeke LJ, Herman MA. Identification and characterization of differentially expressed genes in Caenorhabditis elegans in response to pathogenic and nonpathogenic stenotrophomonas maltophilia. BMC Microbiol. 2020;20(1):170. doi:10.1186/s12866-020-01771-1. 32560629 PMC7304212

[30] Ding W, Smulan LJ, Hou NS, Taubert S, Watts JL, Walker AK. s-Adenosylmethionine levels govern innate immunity through distinct methylation-dependent pathways. Cell Metab. 2015;22(4):633–645. doi:10.1016/j.cmet.2015.07.013. 26321661 PMC4598287

[31] Kishino S, Takeuchi M, Park SB, Hirata A, Kitamura N, Kunisawa J, Kiyono H, Iwamoto R, Isobe Y, Arita M, et al. Polyunsaturated fatty acid saturation by gut lactic acid bacteria affecting host lipid composition. Proc Natl Acad Sci U S A. 2013;110(44):17808–17813. doi:10.1073/pnas.1312937110. 24127592 PMC3816446

[32] Wang X, Li GH, Zou CG, Ji XL, Liu T, Zhao PJ, Liang LM, Xu JP, An ZQ, Zheng X, *et al*. Bacteria can mobilize nematode-trapping fungi to kill nematodes. Nat Commun. 2014;5(1):5776. doi:10.1038/ncomms6776. 25514608 PMC4275587

[33] Liu W, Tian XQ, Wei JW, Ding LL, Qian W, Liu Z, Wang FF. BsmR degrades c-di-GMP to modulate biofilm formation of nosocomial pathogen Stenotrophomonas maltophilia. Sci Rep. 2017;7(1):4665. doi:10.1038/s41598-017-04763-w. 28680041 PMC5498567

[34] Squiban B, Belougne J, Ewbank J, Zugasti O. Quantitative and automated high-throughput genome-wide RNAi screens in C. elegans. J Vis Exp. 2012;(60):3448. doi:10.3791/3448. PMC3399495 22395785

[35] Hills T, Brockie PJ, Maricq AV. Dopamine and glutamate control area-restricted search behavior in Caenorhabditis elegans. J Neurosci. 2004;24:1217–1225 doi:10.1523/JNEUROSCI.1569-03.2004. 14762140 PMC6793574

[36] Meex RC, Schrauwen P, Hesselink MK. Modulation of myocellular fat stores: lipid droplet dynamics in health and disease. Am J Physiol Regul Integr Comp Physiol. 2009;297(4):R913–924. doi:10.1152/ajpregu.91053.2008. 19692657

[37] Khor VK, Shen WJ, Kraemer FB. Lipid droplet metabolism. Curr Opin Clin Nutr Metab Care. 2013;16(6):632–637. doi:10.1097/MCO.0b013e3283651106. 24100667 PMC4006541

[38] Liu Z, Li X, Ge Q, Ding M, Huang X. A lipid droplet-associated GFP reporter-based screen identifies new fat storage regulators in C. elegans. J Genet Genomics. 2014;41(5):305–313. doi:10.1016/j.jgg.2014.03.002. 24894357

[39] Xie M, Roy R. The Causative Gene in Chanarian Dorfman Syndrome Regulates Lipid Droplet Homeostasis in C. elegans. PLoS Genet. 2015;11(6):e1005284. doi:10.1371/journal.pgen.1005284. 26083785 PMC4470697

[40] Lin C, Lin Y, Meng T, Lian J, Liang Y, Kuang Y, Cao Y, Chen Y. Anti-fat effect and mechanism of polysaccharide-enriched extract from Cyclocarya paliurus (Batal.) Iljinskaja in Caenorhabditis elegans. Food Funct. 2020;11(6):5320–5332. doi:10.1039/C9FO03058A. 32458846

[41] Yang F, Vought BW, Satterlee JS, Walker AK, Jim Sun ZY, Watts JL, DeBeaumont R, Saito RM, Hyberts SG, Yang S, *et al*. An ARC/Mediator subunit required for SREBP control of cholesterol and lipid homeostasis. Nature. 2006;442(7103):700–704. doi:10.1038/nature04942. 16799563

[42] Walker AK, Jacobs RL, Watts JL, Rottiers V, Jiang K, Finnegan DM, Shioda T, Hansen M, Yang F, Niebergall LJ, *et al*. A conserved SREBP-1/phosphatidylcholine feedback circuit regulates lipogenesis in metazoans. Cell. 2011;147(4):840–852. doi:10.1016/j.cell.2011.09.045. 22035958 PMC3384509

[43] Shi X, Li J, Zou X, Greggain J, Rodkaer SV, Faergeman NJ, Liang B, Watts JL. Regulation of lipid droplet size and phospholipid composition by stearoyl-CoA desaturase. J Lipid Res. 2013;54:2504–2514. 23787165 10.1194/jlr.M039669 PMC3735947

[44] Sampath H, Miyazaki M, Dobrzyn A, Ntambi JM. Stearoyl-CoA desaturase-1 mediates the pro-lipogenic effects of dietary saturated fat. J Biol Chem. 2007;282(4):2483–2493. doi:10.1074/jbc.M610158200. 17127673

[45] Miyazaki M, Flowers MT, Sampath H, Chu K, Otzelberger C, Liu X, Ntambi JM. Hepatic stearoyl-CoA desaturase-1 deficiency protects mice from carbohydrate-induced adiposity and hepatic steatosis. Cell Metab. 2007;6(6):484–496. doi:10.1016/j.cmet.2007.10.014. 18054317

[46] Miyazaki M, Dobrzyn A, Man WC, Chu K, Sampath H, Kim HJ, Ntambi JM. Stearoyl-CoA desaturase 1 gene expression is necessary for fructose-mediated induction of lipogenic gene expression by sterol regulatory element-binding protein-1c-dependent and -independent mechanisms. J Biol Chem. 2004;279(24):25164–25171. doi:10.1074/jbc.M402781200. 15066988

[47] Kim HJ, Miyazaki M, Ntambi JM. Dietary cholesterol opposes PUFA-mediated repression of the stearoyl-CoA desaturase-1 gene by SREBP-1 independent mechanism. J Lipid Res. 2002;43(10):1750–1757. doi:10.1194/jlr.M100433-JLR200. 12364560

[48] Wu J, Jiang X, Li Y, Zhu T, Zhang J, Zhang Z, Zhang L, Zhang Y, Wang Y, Zou X, *et al*. PHA-4/FoxA senses nucleolar stress to regulate lipid accumulation in Caenorhabditis elegans. Nat Commun. 2018;9(1):1195. doi:10.1038/s41467-018-03531-2. 29567958 PMC5864837

[49] Butcher RA, Ragains JR, Li W, Ruvkun G, Clardy J, Mak HY. Biosynthesis of the Caenorhabditis elegans dauer pheromone. Proc Natl Acad Sci U S A. 2009;106(6):1875–1879. doi:10.1073/pnas.0810338106. 19174521 PMC2631283

[50] Zhang SO, Box AC, Xu N, Le Men J, Yu J, Guo F, Trimble R, Mak HY. Genetic and dietary regulation of lipid droplet expansion in Caenorhabditis elegans. Proc Natl Acad Sci U S A. 2010;107(10):4640–4645. doi:10.1073/pnas.0912308107. 20176933 PMC2842062

[51] Fagone P, Jackowski S. Membrane phospholipid synthesis and endoplasmic reticulum function. J Lipid Res. 2009;50:S311–316. doi:10.1194/jlr.R800049-JLR200. 18952570 PMC2674712

[52] Sorger D, Daum G. Triacylglycerol biosynthesis in yeast. Appl Microbiol Biotechnol. 2003;61(4):289–299. doi:10.1007/s00253-002-1212-4. 12743757

[53] Prinz WA. A bridge to understanding lipid droplet growth. Dev Cell. 2013;24(4):335–336. doi:10.1016/j.devcel.2013.02.004. 23449466 PMC3751170

[54] Cao Z, Hao Y, Fung CW, Lee YY, Wang P, Li X, Xie K, Lam WJ, Qiu Y, Tang BZ, *et al*. Dietary fatty acids promote lipid droplet diversity through seipin enrichment in an ER subdomain. Nat Commun. 2019;10(1):2902. doi:10.1038/s41467-019-10835-4. 31263173 PMC6602954

[55] Grippa A, Buxo L, Mora G, Funaya C, Idrissi FZ, Mancuso F, Gomez R, Muntanya J, Sabido E, Carvalho P. The seipin complex Fld1/Ldb16 stabilizes ER-lipid droplet contact sites. J Cell Biol. 2015;211(4):829–844. doi:10.1083/jcb.201502070. 26572621 PMC4657162

[56] Wang H, Becuwe M, Housden BE, Chitraju C, Porras AJ, Graham MM, Liu XN, Thiam AR, Savage DB, Agarwal AK, *et al*. Seipin is required for converting nascent to mature lipid droplets. Elife. 2016;5. doi:10.7554/eLife.16582 PMC5035145 27564575

[57] Salo VT, Belevich I, Li S, Karhinen L, Vihinen H, Vigouroux C, Magre J, Thiele C, Holtta-Vuori M, Jokitalo E, *et al*. Seipin regulates ER-lipid droplet contacts and cargo delivery. EMBO J. 2016;35(24):2699–2716. doi:10.15252/embj.201695170. 27879284 PMC5167346

[58] Pagac M, Cooper DE, Qi Y, Lukmantara IE, Mak HY, Wu Z, Tian Y, Liu Z, Lei M, Du X, *et al*. SEIPIN regulates lipid droplet expansion and adipocyte development by modulating the activity of glycerol-3-phosphate acyltransferase. Cell Rep. 2016;17(6):1546–1559. doi:10.1016/j.celrep.2016.10.037. 27806294 PMC5647143

[59] Yan R, Qian H, Lukmantara I, Gao M, Du X, Yan N, Yang H. Human SEIPIN Binds Anionic Phospholipids. Dev Cell. 2018;47(2):248–256 e244. doi:10.1016/j.devcel.2018.09.010. 30293840

[60] Sui X, Arlt H, Brock KP, Lai ZW, DiMaio F, Marks DS, Liao M, Farese RV Jr., Walther TC. Cryo-electron microscopy structure of the lipid droplet-formation protein seipin. J Cell Biol. 2018;217(12):4080–4091. doi:10.1083/jcb.201809067. 30327422 PMC6279392

[61] Xu N, Zhang SO, Cole RA, McKinney SA, Guo F, Haas JT, Bobba S, Farese RV Jr., Mak HY. The FATP1-DGAT2 complex facilitates lipid droplet expansion at the ER-lipid droplet interface. J Cell Biol. 2012;198(5):895–911. doi:10.1083/jcb.201201139. 22927462 PMC3432760

[62] Jin Y, McFie PJ, Banman SL, Brandt C, Stone SJ. Diacylglycerol acyltransferase-2 (DGAT2) and monoacylglycerol acyltransferase-2 (MGAT2) interact to promote triacylglycerol synthesis. J Biol Chem. 2014;289(41):28237–28248. doi:10.1074/jbc.M114.571190. 25164810 PMC4192479

[63] Li C, Luo X, Zhao S, Siu GK, Liang Y, Chan HC, Satoh A, Yu SS. COPI-TRAPPII activates Rab18 and regulates its lipid droplet association. EMBO J. 2017;36(4):441–457. doi:10.15252/embj.201694866. 28003315 PMC5694949

[64] Xu D, Li Y, Wu L, Li Y, Zhao D, Yu J, Huang T, Ferguson C, Parton RG, Yang H, *et al*. Rab18 promotes lipid droplet (LD) growth by tethering the ER to LDs through SNARE and NRZ interactions. J Cell Biol. 2018;217(3):975–995. doi:10.1083/jcb.201704184. 29367353 PMC5839781

[65] Jayson CBK, Arlt H, Fischer AW, Lai ZW, Farese RV Jr., Walther TC, Barr FA. Rab18 is not necessary for lipid droplet biogenesis or turnover in human mammary carcinoma cells. Mol Biol Cell. 2018;29(17):2045–2054. doi:10.1091/mbc.E18-05-0282. 29949452 PMC6232964

[66] Tan MW, Mahajan-Miklos S, Ausubel FM. Killing of Caenorhabditis elegans by Pseudomonas aeruginosa used to model mammalian bacterial pathogenesis. Proc Natl Acad Sci U S A. 1999;96(2):715–720. doi:10.1073/pnas.96.2.715. 9892699 PMC15202

[67] Kim DH, Feinbaum R, Alloing G, Emerson FE, Garsin DA, Inoue H, Tanaka-Hino M, Hisamoto N, Matsumoto K, Tan MW, *et al*. A conserved p38 MAP kinase pathway in Caenorhabditis elegans innate immunity. Science. 2002;297(5581):623–626. doi:10.1126/science.1073759. 12142542

[68] Irazoqui JE, Ausubel FM. 99th Dahlem conference on infection, inflammation and chronic inflammatory disorders: caenorhabditis elegans as a model to study tissues involved in host immunity and microbial pathogenesis. Clin Exp Immunol. 2010;160(1):48–57. doi:10.1111/j.1365-2249.2010.04122.x. 20415851 PMC2841835

[69] Wani KA, Goswamy D, Irazoqui JE. Nervous system control of intestinal host defense in C. elegans. Curr Opin Neurobiol. 2019;62:1–9. doi:10.1016/j.conb.2019.11.007. 31790812 PMC7255937

[70] Meisel JD, Kim DH. Behavioral avoidance of pathogenic bacteria by Caenorhabditis elegans. Trends Immunol. 2014;35(10):465–470. doi:10.1016/j.it.2014.08.008. 25240986

[71] Singh J, Aballay A. Neural control of behavioral and molecular defenses in C. elegans. Curr Opin Neurobiol. 2019;62:34–40. doi:10.1016/j.conb.2019.10.012. 31812835 PMC7272302

[72] Troemel ER, Chu SW, Reinke V, Lee SS, Ausubel FM, Kim DH. p38 MAPK regulates expression of immune response genes and contributes to longevity in C. elegans. PLoS Genet. 2006;2(11):e183. doi:10.1371/journal.pgen.0020183. 17096597 PMC1635533

[73] Fletcher M, Tillman EJ, Butty VL, Levine SS, Kim DH, Garsin DA. Global transcriptional regulation of innate immunity by ATF-7 in C. elegans. PLoS Genet. 2019;15(2):e1007830. doi:10.1371/journal.pgen.1007830. 30789901 PMC6400416

[74] Kim DH. Signaling in the innate immune response. WormBook. 2018;2018:1–35. doi:10.1895/wormbook.1.83.2. PMC6369418 26694508

[75] Crossman LC, Gould VC, Dow JM, Vernikos GS, Okazaki A, Sebaihia M, Saunders D, Arrowsmith C, Carver T, Peters N, *et al*. The complete genome, comparative and functional analysis of Stenotrophomonas maltophilia reveals an organism heavily shielded by drug resistance determinants. Genome Biol. 2008;9(4):R74. doi:10.1186/gb-2008-9-4-r74. 18419807 PMC2643945

[76] Rohlfing AK, Miteva Y, Hannenhalli S, Lamitina T, Kaeberlein M. Genetic and physiological activation of osmosensitive gene expression mimics transcriptional signatures of pathogen infection in C. elegans. PLoS One. 2010;5(2):e9010. doi:10.1371/journal.pone.0009010. 20126308 PMC2814864

[77] van der Veen JN, Kennelly JP, Wan S, Vance JE, Vance DE, Jacobs RL. The critical role of phosphatidylcholine and phosphatidylethanolamine metabolism in health and disease. Biochim Biophys Acta Biomembr. 2017;185(9):1558–1572. 9 Pt B 10.1016/j.bbamem.2017.04.006 28411170

[78] Zhang J, Hu Y, Wang Y, Fu L, Xu X, Li C, Xu J, Li C, Zhang L, Yang R, *et al*. mmBCFA C17iso ensures endoplasmic reticulum integrity for lipid droplet growth. J Cell Biol. 2021;220(11). doi:10.1083/jcb.202102122. PMC8563294 34623380

[79] Kemphues KJ, Kusch M, Wolf N. Maternal-effect lethal mutations on linkage group II of Caenorhabditis elegans. Genetics. 1988;120(4):977–986. doi:10.1093/genetics/120.4.977. 3224814 PMC1203589

[80] Encalada SE, Martin PR, Phillips JB, Lyczak R, Hamill DR, Swan KA, Bowerman B. DNA replication defects delay cell division and disrupt cell polarity in early Caenorhabditis elegans embryos. Dev Biol. 2000;228(2):225–238. doi:10.1006/dbio.2000.9965. 11112326

[81] Davis MW, Hammarlund M, Harrach T, Hullett P, Olsen S, Jorgensen EM. Rapid single nucleotide polymorphism mapping in C. elegans. BMC Genomics. 2005;6(1):118. doi:10.1186/1471-2164-6-118. 16156901 PMC1242227

[82] Ding Y, Zhang S, Yang L, Na H, Zhang P, Zhang H, Wang Y, Chen Y, Yu J, Huo C, *et al*. Isolating lipid droplets from multiple species. Nat Protoc. 2013;8(1):43–51. doi:10.1038/nprot.2012.142. 23222457

[83] Weisenfeld NI, Kumar V, Shah P, Church DM, Jaffe DB. Direct determination of diploid genome sequences. Genome Res. 2017;27(5):757–767. doi:10.1101/gr.214874.116. 28381613 PMC5411770

[84] Dillin A, Crawford DK, Kenyon C. Timing requirements for insulin/IGF-1 signaling in C. elegans. Science (New York, NY). 2002;298(5594):830–834. doi:10.1126/science.1074240. 12399591

[85] Apfeld J, Kenyon C. Regulation of lifespan by sensory perception in Caenorhabditis elegans. Nature. 1999;402(6763):804–809. doi:10.1038/45544. 10617200

[86] Tawe WN, Eschbach ML, Walter RD, Henkle-Duhrsen K. Identification of stress-responsive genes in Caenorhabditis elegans using RT-PCR differential display. Nucleic Acids Res. 1998;26(7):1621–1627. doi:10.1093/nar/26.7.1621. 9512531 PMC147444

[87] Brock TJ, Browse J, Watts JL. Fatty acid desaturation and the regulation of adiposity in Caenorhabditis elegans. Genetics. 2007;176:865–875. 17435249 10.1534/genetics.107.071860 PMC1894614

[88] Raizen DM, Lee RY, Avery L. Interacting genes required for pharyngeal excitation by motor neuron MC in Caenorhabditis elegans. Genetics. 1995;141(4):1365–1382. doi:10.1093/genetics/141.4.1365. 8601480 PMC1206873

[89] Zhu H, Sewell AK, Han M. Intestinal apical polarity mediates regulation of TORC1 by glucosylceramide in C. elegans. Genes Dev. 2015;29(12):1218–1223. doi:10.1101/gad.263483.115. 26109047 PMC4495394

[90] Li X, Ji G, Chen X, Ding W, Sun L, Xu W, Han H, Sun F. Large scale three-dimensional reconstruction of an entire Caenorhabditis elegans larva using AutoCUTS-SEM. J Struct Biol. 2017;200(2):87–96. doi:10.1016/j.jsb.2017.09.010. 28978429

