# Supplementary material for: Dietary S. maltophilia induces supersized lipid droplets by enhancing lipogenesis and ER-LD contacts in C. elegans
Source: Gut Microbes. 2022 Feb 3;14(1):2013762. doi: 10.1080/19490976.2021.2013762 (PMC8816401; doi:10.1080/19490976.2021.2013762)
Supplement: Supplemental Material [file KGMI_A_2013762_SM6206.docx]

**Supporting Information
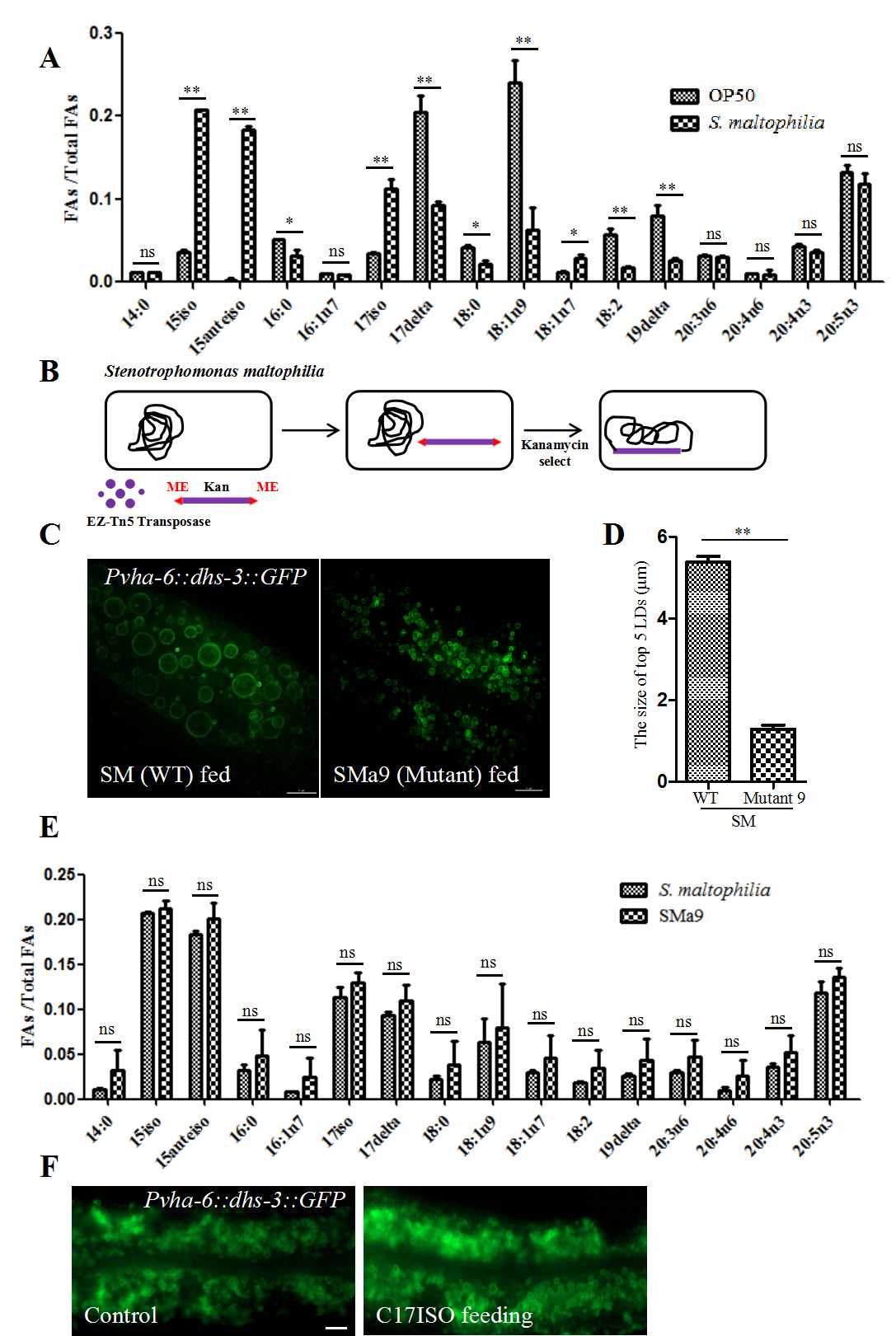
**

**Figure S1 Differential fatty acids were not the cause for the enlargement of LDs in *S. maltophilia*-fed nematode.**

**(A)** The abundance of all fatty acids detected in OP50 and *S. maltophilia* strain. Mean ± SEM from three independent samples is shown. The assay was repeated three times, student’s t-test and one-way ANOVA, 0.01<**P*<0.05, *** *P*<0.001, ns, no significance. **(B)** The cartoon to show the method to obtain the bacterial mutants. **(C)** Fluorescence micrographs of *Pvha-6::dhs-3::GFP* in *S. maltophilia* (WT) and *S. maltophilia* (SMa9)*-*fed worms. Scale bar = 5 μm. **(D)** Distribution of the LD size (% lipid droplets) for (C). Data are presented as mean ± SD of 10 animals for each worm strain, the assay was repeated three times, student’s *t*-test, 0.01<**P*<0.05, *** *P*<0.001, ns, no significance. **(E)** The abundance of all fatty acids detected in *S. maltophilia* strain and *S. maltophilia* SMa9. The assay was repeated three times, student’s t-test and one-way ANOVA, ns, no significance. **(F)** Worms at L1 stage were fed with OP50 in the presence or absence of C17iso for 48 h and examined using fluorescence microscope. Scale bar = 5 μm.

**
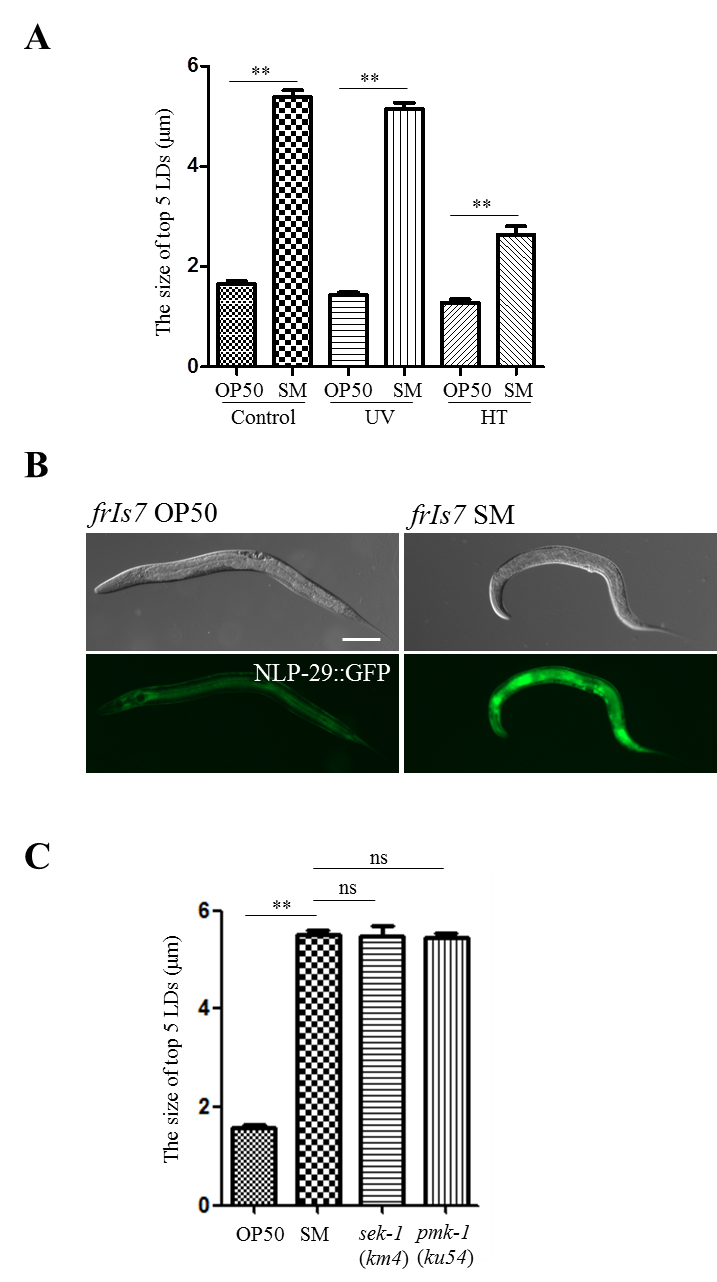
**

**Figure S2 *S. maltophilia* increases NLP-29 protein level in *C. elegans.***

**(A**) Quantification of the LD diameter in N2 animals after feeding with untreated*,* UV-killed*,* high temperature-killed OP50 and *S. maltophilia*. For this statistic, the size of top 5 LDs was analyzed to represent the change in LD diameter. Data are presented as mean ± SD of 15 animals for each worm strain, the assay was repeated three times, student’s *t*-test, ***P*<0.01. **(B)** *frIs7* strain worms were fed with OP50 or *S. maltophilia* from L1 to L4 stage. The protein expression level is shown by fluorescence images. Scale bar = 100 μm. **(C)** Quantification of the LD diameter in *sek-1*(*km4*) and *pmk-1*(*ku54*) animals after feeding with *S. maltophilia*. Data are presented as mean ± SD of 15 animals for each worm strain, the assay was repeated three times, student’s *t*-test, ** *P*<0.01, ns, no significance.


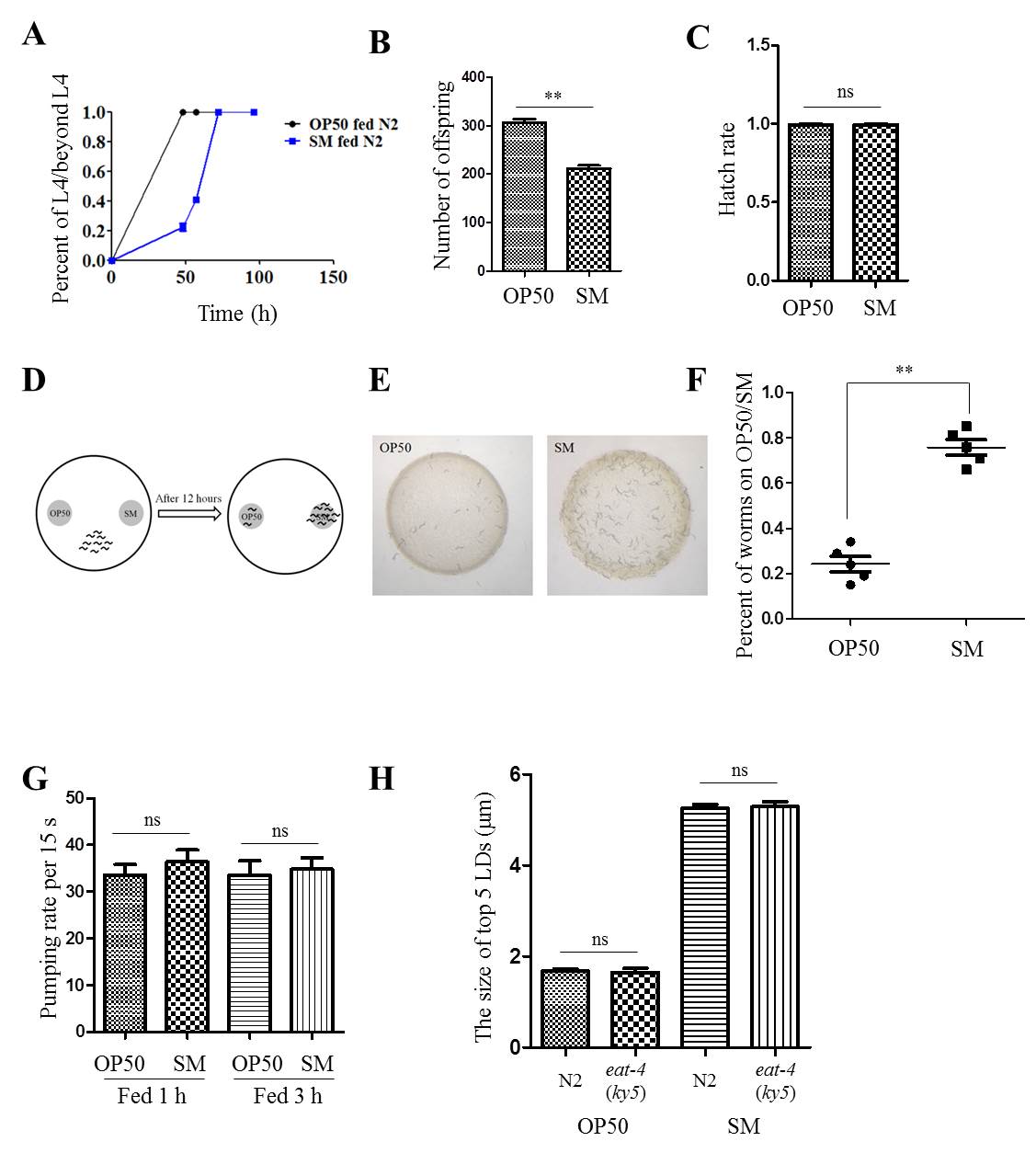


**Figure S3 Physiological measurements in *C. elegans* fed OP50 or *S. maltophilia.***

**(A)** Statistics of growth rate in OP50 and *S. maltophilia*-fed N2. **(B)** Statistics of number of offspring in OP50 and *S. maltophilia*-fed N2. Data are presented as mean ± SD of 15 animals for each worm strain, the assay was repeated three times, student’s *t*-test, ** *P*<0.01. **(C)**. Statistics of hatch rate in OP50 and *S. maltophilia*-fed N2. Data are presented as mean ± SD of 15 animals for each worm strain, the assay was repeated three times, student’s *t*-test, ns, no significance. **(D)** Schematic representation of the assay for the selectivity of nematodes for different diets. **(E)** The results of the selectivity of worms for OP50 or *S. maltophilia.* **(F)** Quantification of the selectivity of nematodes for OP50 or *S. maltophilia.* Data are presented as mean ± SD of 100 animals for independent experiment, the assay was repeated three times, student *t*-test, ***P*<0.01. **(G)** Quantification of the pumping rate of N2 worms after OP50 or *S. maltophilia* feeding for 1 h and 3 h. Data are presented as mean ± SD of 15 animals for each worm strain, the assay was repeated three times, student’s *t*-test, ns, no significance. **(H)** Quantification of the diameter of LDs. For this statistic, the size of top 5 LDs was analyzed to represent the change in LD diameter. Data are presented as mean ± SD of 15 animals for each worm strain, the assay was repeated three times, student’s *t*-test, ns, no significance.


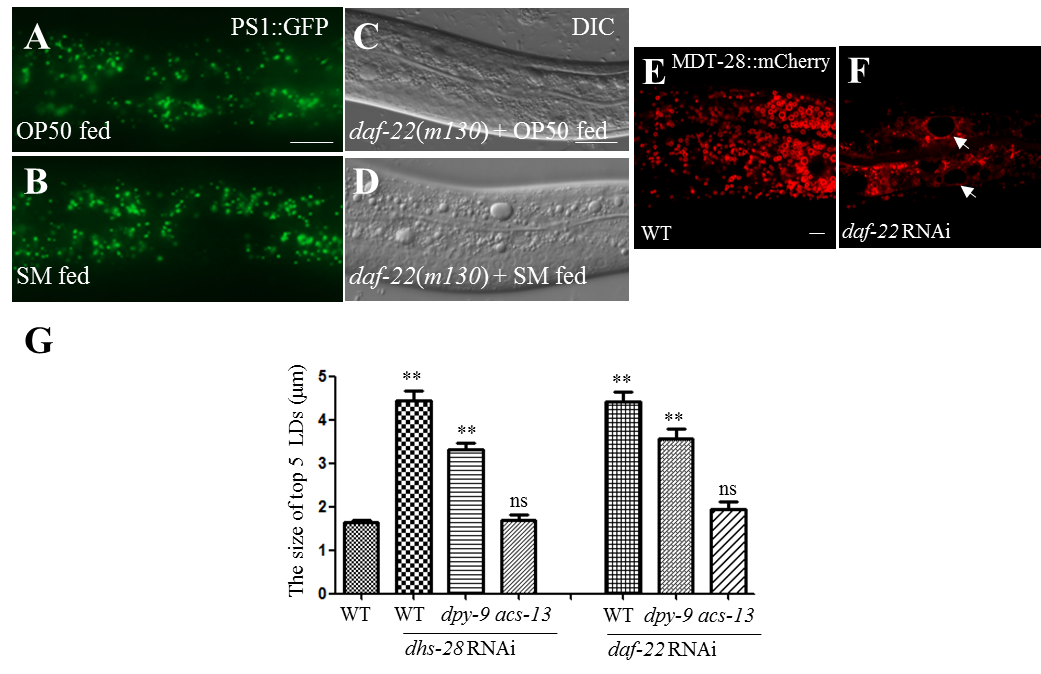


**Figure S4 DPY-9 is specific host factors that are required for dietary *S. maltophilia* to promote LD expansion.**

**(A)** Fluorescence micrographs of PS1::GFP labeled peroxisome in OP50-fed worms. Scale bar = 5 μm. **(B)** Fluorescence micrographs of PS1::GFP labeled peroxisome in *S. maltophilia-*fed worms. **(C)** DIC images of LDs in *daf-22*(*m130*) mutant animals fed OP50. Scale bar = 5 μm. **(D)** DIC images of LDs in *daf-22*(*m130*) mutant animals fed *S. maltophilia*. **(E)** Fluorescence micrographs of MDT-28::mCherry labeled LDs in OP50-fed worms. Scale bar = 5 μm. **(F)** Fluorescence micrographs of MDT-28::mCherry labeled LDs in *daf-22* RNAi animals. The white arrows point to the enlarged LDs. **(G)** Quantification of LD diameter for the results of *Pmdt-28::mdt-28::mCherry* (Control), *dpy-5*(*e61*, Negative control), *dpy-9*(*e12*), and *acs-13*(*vc2046*), worms fed with HT115 in the presence or absence of *daf-22* RNAi and *dhs-28* RNAi. Data are presented as mean ± SD of 15 animals for each worm strain, the assay was repeated three times, student’s *t*-test, ***P*<0.01, ns, no significance.

**
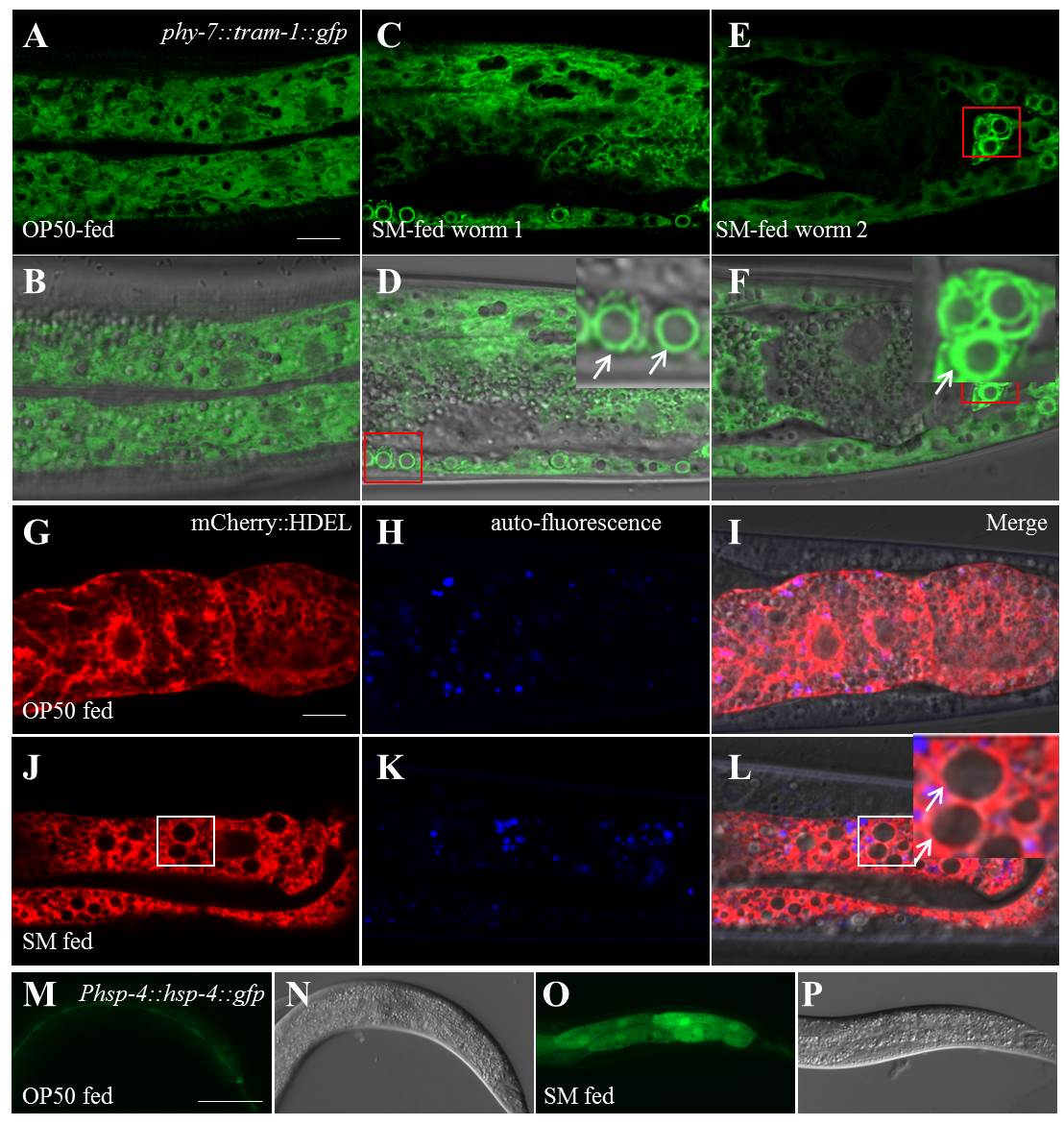
**

**Figure S5 *S. maltophilia* induces the ER association with LDs and ER stress.**

**(A)** Fluorescence micrographs of *Phyp-7::tram-1::gfp* in OP50 fed worms. Scale bar = 5 μm. **(B)** As in (A), but merged with DIC. **(C)** Fluorescence micrographs of *Phyp-7::tram-1::gfp* in *S. maltophilia-*fed worms. **(D)** As in (C), but merged with DIC. **(E)** As in (C), the repeated experiment results. **(F)** As in (D), the repeated experiment results. **(G)** Fluorescence micrographs of mCherry::HDEL in OP50 fed worms. Scale bar = 5 μm. **(H)** Fluorescence micrographs of auto-fluorescence in OP50 fed worms. **(I)** As in (G), but with (H) and DIC merged. **(J)** Fluorescence micrographs of mCherry::HDEL in *S. maltophilia*-fed worms. **(K)** Fluorescence micrographs of auto-fluorescence in *S. maltophilia*-fed worms. **(L)** As in (J), but with (K) and DIC merged. **(M)** Fluorescence micrographs of *Phsp-4::hsp-4::gfp* in OP50-fed worms. Scale bar = 5 μm. **(N)** DIC images of LDs in OP50-fed worms. **(O)** Fluorescence micrographs of *Phsp-4::hsp-4::gfp* in *S. maltophilia*-fed worms. **(P)** DIC images of LDs in *S. maltophilia*-fed worms.


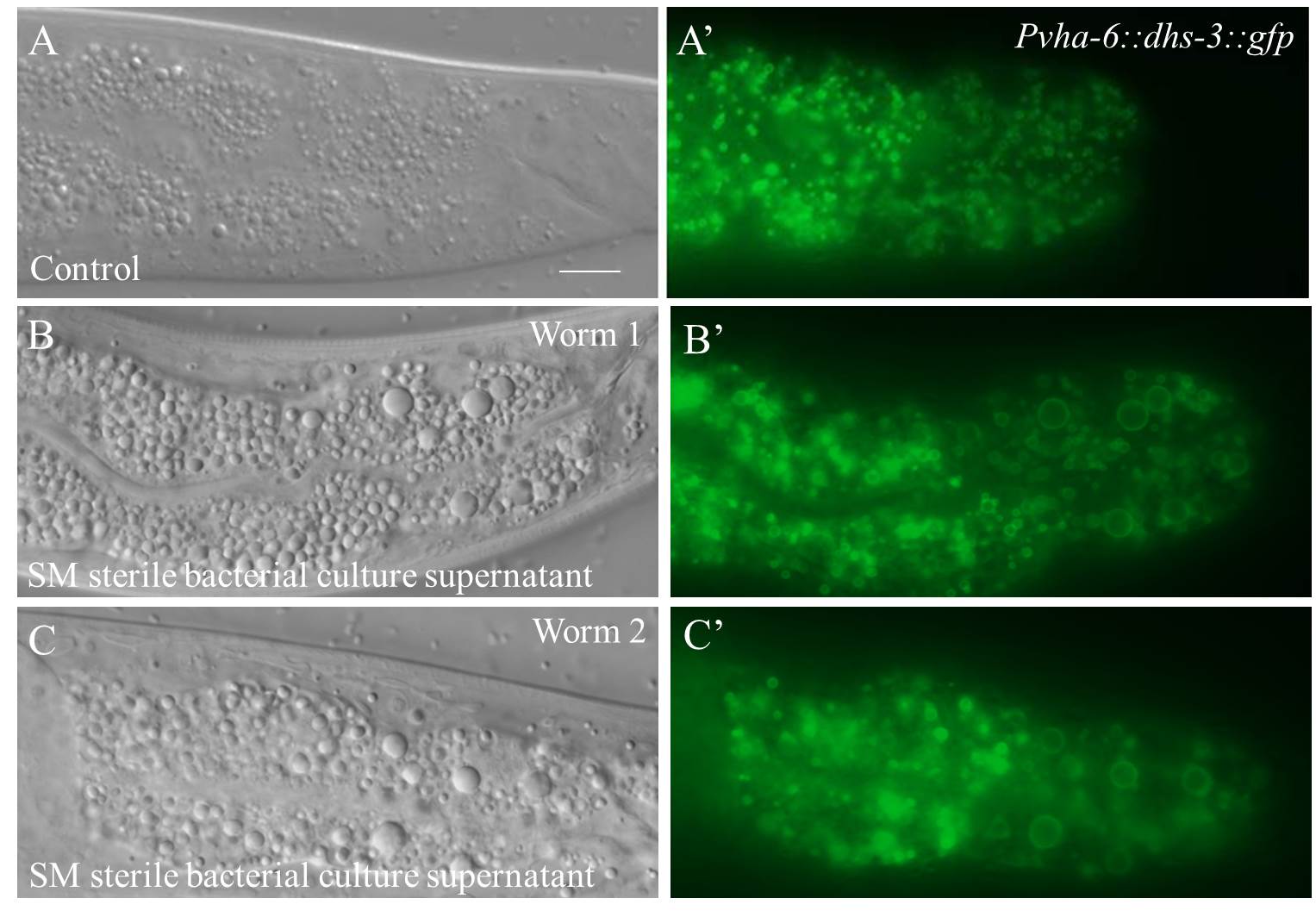


**Figure S6 Culture supernatant of *S. maltophilia* slightly induces large lipid droplets.**

Worms at L1 stage were fed with OP50 in the presence or absence of *S. maltophilia* sterile bacterial culture supernatant for 2.5 days and examined using fluorescence microscope. **(A-C)** DIC images of posterior of worms *Pvha-6::dhs-3::GFP* with (B and C) and without (A) *S. maltophilia* sterile bacterial culture supernatant. **(A’-C’)** Fluorescence images of posterior of worms *Pvha-6::dhs-3::GFP* with (B and C) and without (A) *S. maltophilia* sterile bacterial culture supernatant. Scale bar = 5 μm. SM: *S. maltophilia*.


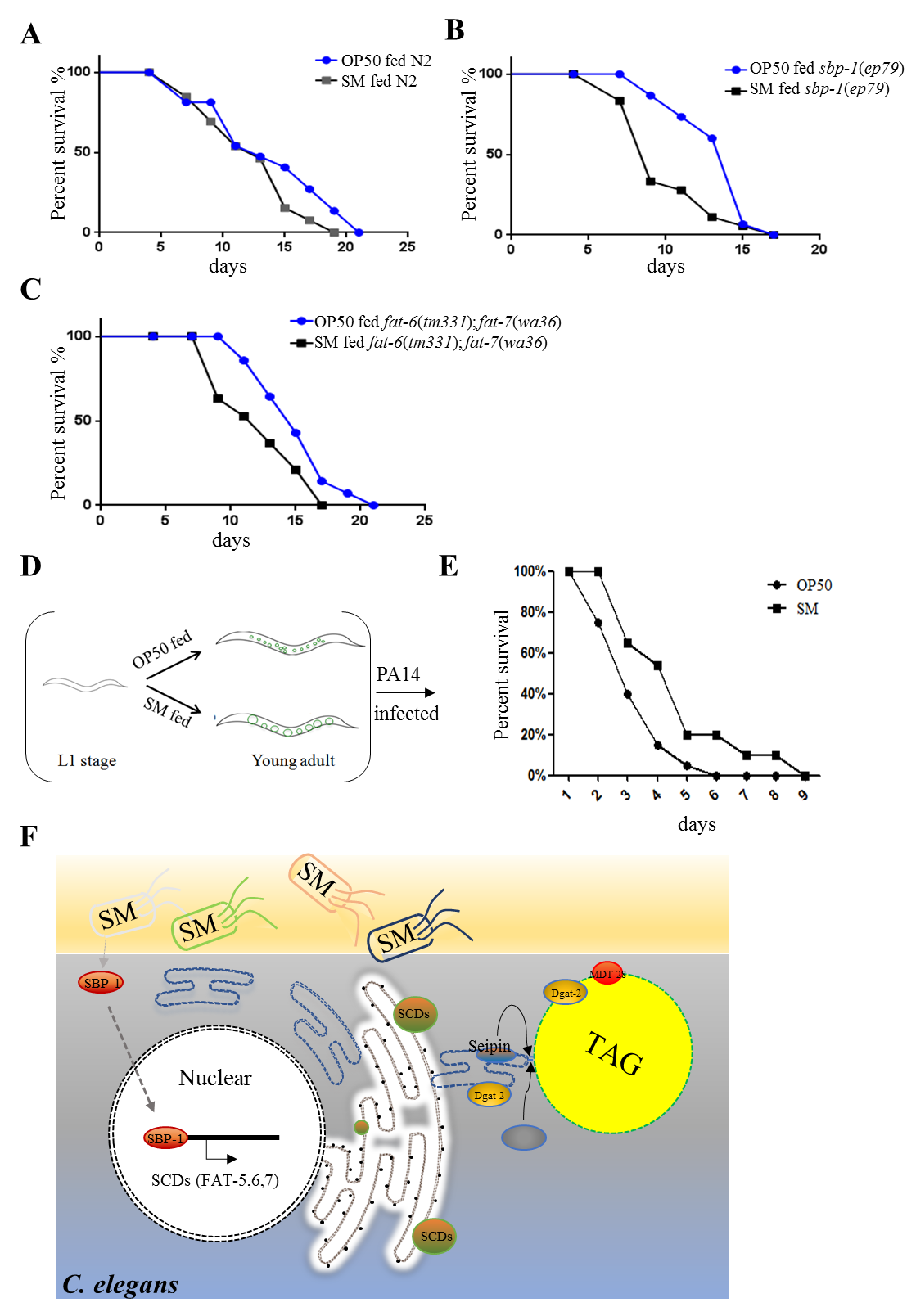


**Figure S7 Enlarged LD formation enhances survival rate.**

**(A)** The percent survival of N2 worms by OP50 and *S. maltophilia* feeding. **(B)** The percent survival of *sbp-1*(*ep79*) strains by OP50 and *S. maltophilia* feeding. **(C)** The percent survival of *fat-6*(*tm331*); *fat-7*(*wa36*) double mutant animals by OP50 and *S. maltophilia* feeding. **(D)** Schematic representation of the assay for (E). The N2 worms were used to feed the OP50 and *S. maltophilia* from L1 to L4 stage, then the OP50 and *S. maltophilia-*fed worms were picked on PA14 plate, then the percent survival of those worms was quantified. **(E)** The percent survival of OP50 and *S. maltophilia-*fed worms by PA14 infection. SM: *S. maltophilia*.


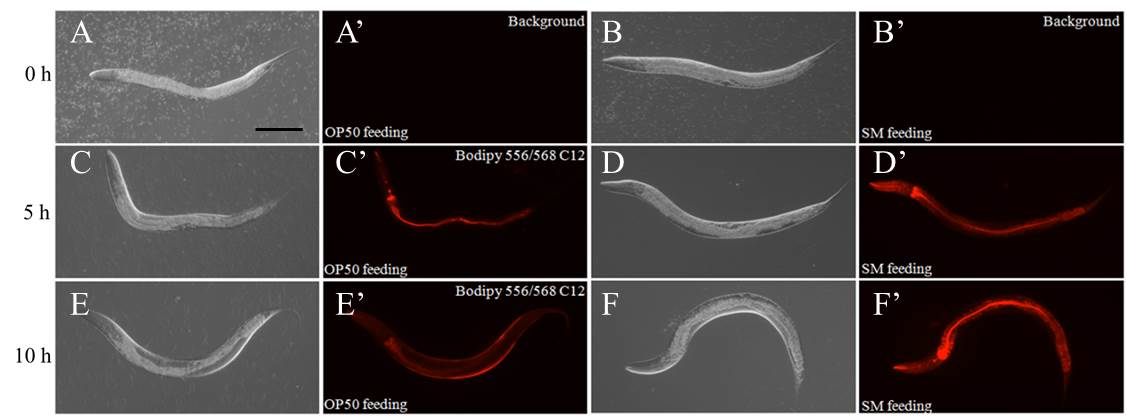


**Figure S8 No significant effect on free fatty acid uptake and incorporation between OP50- and *S. maltophilia*-fed nematodes.**

Worms at **L4** stage were fed with OP50 and *S. maltophilia* respectively for 0, 5, and 10 h, and followed by examination using fluorescence microscope. **(A-F)** DIC images of whole worm fed with OP50 (A, C, E) and *S. maltophilia* (B, D, F) for 0 h (A and B), 5 h (C and D), and 10 h (E and F). **(A’-F’)** Fluorescence images of whole worm fed with OP50 (A’, C’, E’) and *S. maltophilia* (B’, D’, F’) for 0 h (A’ and B’, represent autofluorescence/background), 5 h (C’ and D’), and 10 h (E’ and F’). SM: *S. maltophilia*. Scale bar = 100 μm.


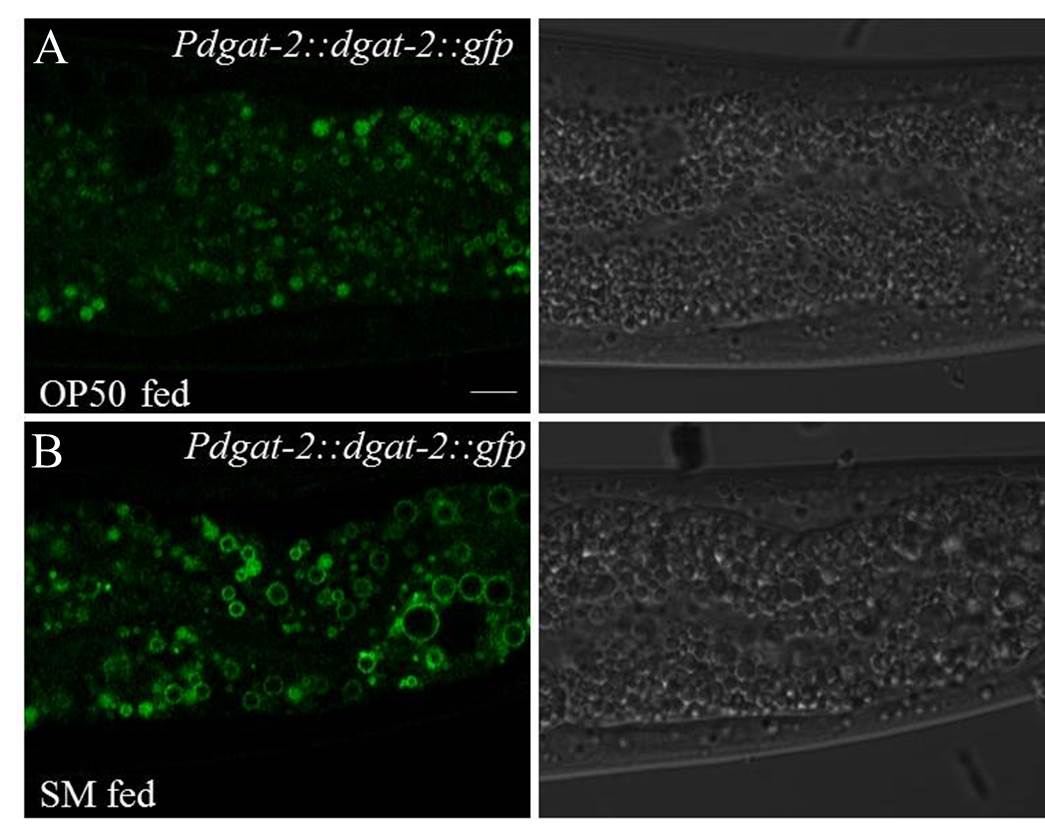


**Figure S9 *S. maltophilia* does not affect the LD positioning of DGAT-2.**

**(A)** Fluorescence image of *Pdgat-2::dgat-2::gfp* in OP50-fed worms. **(B)** as in (A), but with an animal fed with *S. maltophilia*. SM: *S. maltophilia.* Scale bar = 5 μm.


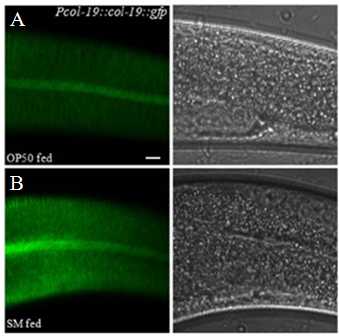


**Figure S10 *S. maltophilia* feeding only increased the fluorescence intensity of COL-19::GFP, but it could not change its texture.**

**(A)** Fluorescence image of *Pcol-19::col-19::gfp* in OP50-fed worms. **(B)** as in (A), but with an animal fed with *S. maltophilia*. SM: *S. maltophilia.* Scale bar = 5 μm.
